# Reprogrammed MDSCs promote Th1‐dominant antitumour response via CD40 induced by autocrine TNF‐α after combining cryo‐thermal therapy with IL6 and IL17A neutralization

**DOI:** 10.1002/ctm2.70493

**Published:** 2025-10-13

**Authors:** Yuankai Hao, Shicheng Wang, Junjun Wang, Zelu Zhang, Yichen Yao, Ke Wang, Ping Liu, Lisa X. Xu

**Affiliations:** ^1^ Med‐X Research Institute School of Biomedical Engineering Shanghai Jiao Tong University Shanghai China

**Keywords:** CD4‐positive T‐lymphocytes, CD40, interleukin‐17A, interleukin‐6, myeloid‐derived suppressor cells, tumour necrosis factor‐alpha (TNF‐α)

## Abstract

**Background:**

Mounting evidence shows that myeloid‐derived suppressor cells (MDSCs) reprogramming can significantly enhance the outcomes of immunotherapy. However, the therapeutic potential of targeting MDSCs alone is limited by persistent immunosuppressive cytokines and cellular crosstalk. In our previous study, we found that novel cryo‐thermal therapy (CTT) can drive MDSCs maturation and induce CD4^+^ T helper type (Th)1‐dominant differentiation, improving long‐term survival in spontaneous high metastatic mouse models. Considering the established roles of Interleukin (IL)‐6 and IL‐17A in non‐small cell lung cancer (NSCLC) progression and immune evasion, we developed a combination strategy integrating cytokine neutralization with CTT (combination therapy) in LLC1 tumor‐bearing mice. Although the combination therapy successfully promoted MDSCs maturation and Th1 differentiation, the underlying mechanistic basis remained unclear.

**Methods:**

The combination therapy was implemented in LLC1 tumor‐bearing mice. We then observed its impacts on MDSCs maturation and Th1 differentiation and explored the related mechanisms by examining various aspects including the expression of CD40, the reactive oxygen species (ROS)‐nuclear factor‐kappa B (NF‐κB) pathway, and the induction of tumor necrosis factor‐α (TNF‐α).

**Results:**

It was observed that the combination therapy increased the expression of CD40 on MDSCs through the ROS‐NF‐κB pathway‐dependent TNF‐α induction. This TNF‐α‐mediated CD40 upregulation facilitated Th1 polarization via CD40L engagement on CD4^+^ T cells. Our results provided the first mechanistic evidence that autocrine TNF‐α production by reprogrammed MDSCs governs CD40 expression following combination therapy.

**Conclusion:**

Our research elucidated the methods and mechanisms of MDSCs reprogramming and offered a promising therapeutic strategy for patients with NSCLC and other types of cancer.

## Graphical abstract

The combination of IL‐6 and IL‐17A neutralizing antibodies and cryo‐thermal therapy promotes the expression of CD40 on MDSCs by inducing TNF‐α production via the NF‐κB pathway in MDSCs. The CD40–CD40L interaction initiates the Th1‐dominant differentiation of CD4^+^ T cells, leading to long‐term antitumour immunity.

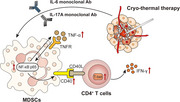

## 1 INTRODUCTION

Recent clinical advances have demonstrated the efficacy of immunotherapies targeting functionally impaired or exhausted T cells, as well as engineered T cell approaches.^1^, ^2^ Nevertheless, the therapeutic potential of these strategies is substantially limited by immunosuppressive cellular components within the tumour microenvironment (TME), particularly myeloid‐derived suppressor cells (MDSCs).^3^ MDSCs actively suppress T cell activation and cytotoxic function, thereby facilitating immune evasion in multiple malignancies.^4^ Consequently, therapeutic reprogramming of this immunosuppressive TME represents a critical requirement for achieving optimal antitumour responses.^5^

The cytokine network is fundamental to the orchestration of tumour progression and immune cell interactions in the TME.^6^, ^7^ For example, TGF‐β, IL‐1 and IL‐17A promote tumour cell proliferation and metastasis^8^, ^9^, ^10^; IL‐6 can disrupt cytokine regulation and promote tumour progression^11^; and IL‐4, IL‐10 and TGF‐β cause immunosuppression.^12^, ^13^, ^14^ Recent advances in targeting these cytokines have shown promising therapeutic potential for modulating the immune system, inhibiting tumour progression, and overcoming resistance to conventional therapies. However, most clinical trials of cytokine mAbs have shown either limited or no beneficial effects.^15^ Additionally, single anti‐cytokine therapy fails to remodel the immunosuppressive environment and ultimately leads to tumour progression, which implies that combination strategies have opened new avenues for overcoming resistance to conventional immunotherapies and improving patient outcomes.^16^

In our previous studies, we established a novel physiotherapy for solid tumour ablation in situ by alternating liquid nitrogen cooling followed by radiofrequency (RF) heating called cryo‐thermal therapy (CTT).^17^ CTT promotes the maturation of innate immune cells, including MDSCs,^18^ induces CD4^+^ Th1‐dominant differentiation and ultimately systematically remodels the immune environment,^19^ which efficiently improves the long‐term survival rate of melanoma‐ and triple‐negative breast cancer‐bearing mice.^20^, ^21^ However, in Lewis lung cancer 1 (LLC1)‐bearing mice, CTT administered alone only prolonged the survival time and failed to completely inhibit spontaneous tumour metastasis,^22^ suggesting that we should explore optimal combination therapies for the control of highly malignant tumours.

High levels of IL‐6 and IL‐17A can induce immunosuppression, and several strategies for blocking these two cytokines as therapeutic targets have been proposed.^23^, ^24^ In our recent studies, given that the serum concentrations of IL‐6 and IL‐17A, which are essential cytokines that facilitate non‐small cell lung cancer (NSCLC) progression and MDSCs‐mediated evasion, were markedly increased in LLC1 tumour model after CTT, we combined CTT with IL‐6 and IL‐17A neutralization (combination therapy), which increased the survival rate of an LLC1 mouse model. Mechanistically, combination therapy not only reduces the accumulation of MDSCs but also promotes their maturation, activates CD4^+^ Th1 cell‐mediated antitumour immunity and enhances effective T and NK cell‐mediated tumour killing.^22^ Immunosuppressive MDSCs prevent CD4^+^ T cells from differentiating towards Th1 subtypes and facilitate the polarization of Tregs, resulting in tumour progression.^25^, ^26^ Thus, in our previous study, the maturation of MDSCs and the differentiation of Th1‐dominant CD4^+^ T cells after combination therapy suggested that combination therapy results in remodelling of the systemic immune environment, which in turn contributes to the reciprocal regulation of MDSCs and CD4^+^ T cells. The potential mechanisms of interactions between MDSCs and CD4^+^ T cells after combination therapy remain to be elucidated.

This study demonstrates that combination therapy drives MDSCs maturation and promotes Th1‐polarized CD4^+^ T cell differentiation in the LLC1 model. Through integrated RNA sequencing and in vitro validation, we established that combination therapy‐reprogrammed MDSCs mediate Th1 bias via CD40–CD40L interaction.

Crucially, we identified an autocrine loop wherein MDSCs‐derived TNF‐α upregulates CD40 expression, which in turn reinforces TNF‐α production through NF‐κB pathway activation. These findings reveal a previously unrecognized self‐reinforcing mechanism where mature MDSCs autonomously regulate CD40/TNF‐α expression via NF‐κB signalling, ultimately establishing systemic antitumour immunity characterized by CD4^+^ Th1 dominance.

## 2 MATERIALS AND METHODS

### 2.1 Cell cultivation and mouse tumour model establishing

Lewis lung carcinoma LLC1 cell line was donated by Professor Weiliang Xia, Shanghai Jiao Tong University. Colon cancer MC38 cell line was donated by Professor Hongchen Gu, Shanghai Jiao Tong University. Cells were cultivated in DMEM Dulbecco's Modified Eagle's Medium (HyClone) supplemented with 10% heat‐inactivated fetal bovine serum FBS (GEMINI) and 1% antibiotics (100 U/mL penicillin and 100 µg/mL streptomycin, HyClone).

Female C57BL/6 mice (Slaccas) were housed and fed sterile food with standard mice nutritional formula and sterile water in the isolated cages of 12 h light/dark cycle environment. 1 × 10^6^ LLC1 cells were subcutaneously injected into the right back of female C57BL/6 mice (aged 6–8 weeks) and to establish tumour model. 5 × 10^5^ and 1 × 10^5^ MC38 cells were subcutaneously injected into the right and left back of female C57BL/6 mice, respectively. Mice were euthanized when the humanitarian endpoint was reached. Tumour sizes were monitored every 2 days and its volume was estimated using the following formula: *V* (cm^3^)  =  π × *L* (major axis) × *W* (minor axis) × *H* (vertical axis)/6. The animal study protocol was approved by the Ethics Committee of School of Biomedical Engineering and Med‐X Research Institute, Shanghai Jiao Tong University (No. 2020017). The study design, experimental procedures, husbandry and management, sample size, assignment of animals to experimental groups and experimental results of the animal experiments in this study adhered to ARRIVE guidelines.

### 2.2 The treatment procedures

The cryo‐thermal therapy was performed by successively liquid nitrogen (LN2) freezing at ‐20°C for 5 min and radiofrequency ablation at 50°C for 10 min.

For in vivo IL‐6 and IL‐17A neutralization, mice were intraperitoneally (i.p.) injected with anti‐IL‐6 mAb (MP5‐20F3, 20 µg in 100 µL PBS, Bio X Cell) and anti‐IL‐17A mAb (17F3, 20 µg in 100 µL PBS, Bio X Cell) on day 1, 4, 7, 10, 13 and day 5, 9, 13 post treatment.

For in vivo clearance of ROS, mice treated with the combination therapy were simultaneously injected intraperitoneally with reduced L‐glutathione (GSH, 10 mg/kg, Yeasen) twice daily.

### 2.3 Cell isolation of MDSCs and CD4+ T Cells

The lungs were collected from different groups at indicated times after cryo‐thermal therapy. Single pulmonary cell suspensions were acquired for immunocyte isolation. For MDSCs isolation, GR‐1 positive cells were labelled with APC by monoclone antibody (Biolegend), and subsequently selected with the EasySep Mouse APC Positive Selection Kit II (Cat# 17667, StemCell Technologies) following the given instructions from the manufacturer. To obtain CD4^+^ T cells, EasySep Mouse CD4 Positive Selection Kit II (Cat# 18952, StemCell Technologies) were used.

### 2.4 RNA sequencing and analysis

Total RNA was obtained from isolated MDSCs and CD4^+^ T cells as former description. The purity and quantity of RNA were assessed using the NanoDrop 2000 spectrophotometer (Thermo Scientific), and the integrity was evaluated using Agilent 2100 Bioanalyser (Agilent Technologies). Subsequently, RNA‐seq libraries were generated using the VAHTS Universal V6 RNA‐seq Library Prep Kit. OE Biotech Co., Ltd. was entrusted with conducting subsequent transcriptome sequencing and analysis.

The GSEA software was used to perform enrichment analysis. This analysis involved the utilization of a predefined gene set, where genes were ranked based on their differential expression between the two sample types. The objective was to assess whether there was significant enrichment (Nominal *p*‐value < 0.05, FDR *q*‐value < 0.05, and NES > 1.5) of the predefined gene set at either the upper or lower end of the ranking list. Bioinformatic analysis was performed using the OECloud tools at https://cloud.oebiotech.com.

### 2.5 In vitro immune cells culture assay

To investigate the role and mechanism of MDSCs on CD4^+^ T cell differentiation, CD4^+^ T cells after cryo‐thermal therapy on Day 14 were separated and direct co‐cultured with MDSCs after cryo‐thermal or combination therapy in the ratio of 1:2 with or without CD40L blockade (clone MR‐1, 10 ng/mL, Bio X Cell) and TNF‐α monoclonal antibody (clone XT3.11, 10 ng/mL, Bio X Cell), respectively. CD4^+^ T cells were activated by anti‐CD3a antibody (clone 145‐2C11, 1 µg/mL, Biolegend). 10% of mouse serum from corresponding groups was supplemented to simulate the in vivo environment.

### 2.6 Flow cytometry

To prepare single‐cell suspension, tissues were collected on day 14 after cryo‐thermal therapy. The lungs were physically shredded into segments and enzymolyzed with Collagenase (type I), Hyaluronidase (Yeasen) and Dnase (Solarbio) at 37°C for 40 min. Then lungs were separated manually, and red blood cells were depleted by erythrocyte‐lysing.

Zombie Dye was performed to exclude dead cells before surface marker labelling. True‐Nuclear Transcription Factor Buffer Set was used for transcription factor staining. For intracellular cytokine staining, cells were cultured in the presence of cell activation cocktail (containing PMA (phorbol12‐myristate‐13‐acetate), ionomycin, and protein transport inhibitor (Brefeldin A)) for 4 h at the recommended concentration by the manufacturer. Then cells were fixed and permeabilized with fixation buffer and intracellular staining permeabilization wash buffer according to the manufacturer's instructions. All reagents used are listed in Table S1 and all the fluorochrome‐conjugated monoclonal antibodies used are listed in Table S2. Data were collected using BD FACS Aria II cytometer and processed by FlowJo (version 10.8.2). Gating strategy is shown in Figure S1.

### 2.7 mRNA isolation and real‐time PCR

TRIzol and the PrimeScript RT reagent kit (TaKaRa) were used for RNA extraction and reverse transcription. For measuring the expression of genes, ABI 7900HT sequence detection system (Applied Biosystems), along with SDS 2.4 software, was used for data collection purposes. Results were normalized as GAPDH through ΔΔCt method. The list of all primers used can be found in Table S3.

### 2.8 ELISA

The co‐culture supernatant from different groups were collected, and TNF‐α was detected using the ELISA Kit (Boster Biological Technology) according to the manufacturer's instruction. Each ELISA assay sample represents a pooled serum sample or cell culture supernatant collected from three individual mice.

### 2.9 Western Blotting

To obtain protein lysates, 1× RIPA lysis buffer containing proteinase inhibitor (Thermo Fisher Scientific) was used to lyse cells on ice for 30 min. Anti‐Phospho‐NF‐κB p65 (Ser536) (Cell Signaling Technology, #3031), anti‐NF‐κB p65 (Cell Signaling Technology, #8482), anti‐actin (Santa Cruz, sc‐58673), and HRP‐labelled secondary antibody (EpiZyme) were used in this study. Relative protein expression levels were quantified using Image J software (version Fiji). The Western blot images shown are representative of three independent experiments.

### 2.10 Statistics analysis

All data were presented as mean ± standard deviation (SD). For two groups, two‐sided Student's T‐test was used to analyse the results. For multiple groups, one‐way ANOVA was used to analyse the results. The Kaplan–Meier method and the log‐rank test was used to compare the survival rate of mice. All statistical analyses were performed using GraphPad Prism 9.0.

## 3 RESULTS

### 3.1 Combination therapy improved long‐term survival rate of the mice

**FIGURE 1:**
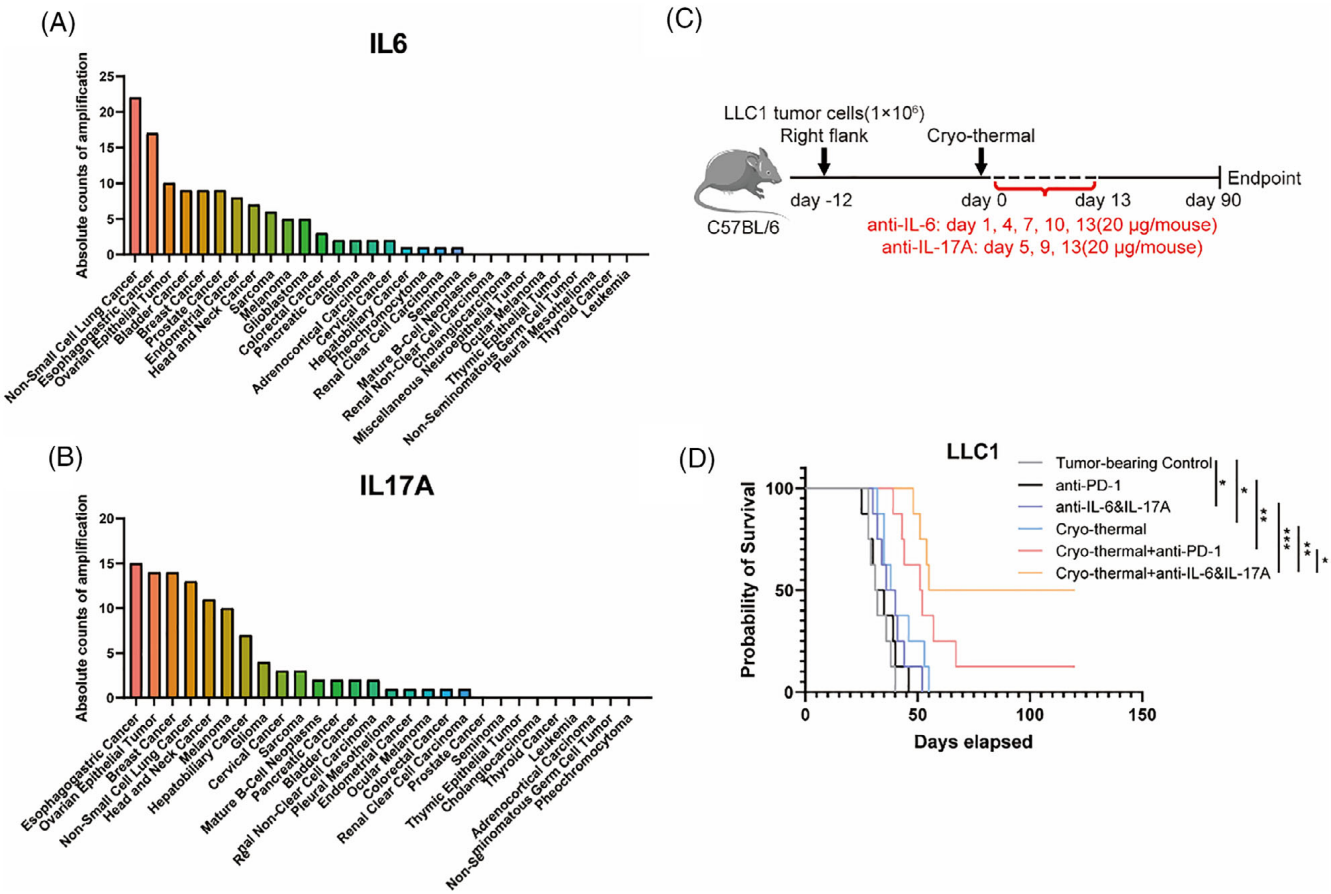
Combination therapy improved long‐term survival rate of the LLC1 mice. (A) The counts of patients with IL6 amplification across cancer types (10 953 patients in the TCGA database). (B) The counts of patients with IL17A amplification across cancer types (10 953 patients in the TCGA database). (C) Scheme of study design. In brief, LLC1 tumour‐bearing mice (*n* = 10/group) were performed cryo‐thermal therapy (tumour‐bearing as control) and intraperitoneally treated with PBS or 20 µg of anti‐IL‐6 and IL‐17A. (D) Kaplan–Meier survival curve of tumour‐bearing control, single treatment of anti‐IL‐6 and IL‐17A, single treatment of anti‐PD‐1, cryo‐thermal therapy, combined anti‐PD‐1 and cryo‐thermal therapy or the combination therapy. The survival curves were compared using log‐rank tests (*n* = 10/group). *p <0.05, **p<0.01, ***p <0.001.

To investigate the association of IL‐6 and IL‐17A with NSCLC, we performed genomic analysis of patient data from The Cancer Genome Atlas (TCGA). Our pan‐cancer screening identified NSCLC as having the highest incidence of *Il6* gene amplifications (*n* = 22 cases, Figure 1A) among all malignancies, along with substantial *Il17a* amplifications (ranking fourth, *n* = 13 cases, Figure 1B). These findings implicate elevated *Il6/Il17a* expression as potential drivers of NSCLC pathogenesis.

To validate the therapeutic efficacy, we employed an LLC1 mouse model following the treatment protocol shown in Figure 1C. Individual treatments consisting of IL‐6/IL‐17A neutralization or CTT alone prolonged mouse survival time but did not increase survival rates compared to tumour‐bearing controls (n = 10 per group, Figure 1D). Remarkably, the combination therapy achieved a 50% long‐term survival rate. Furthermore, although CTT combined with anti‐PD‐1 treatment showed greater survival extension than anti‐PD‐1 monotherapy, its effectiveness remained significantly lower than the combination therapy (Figure 1D). Taken together, these results reconfirmed that the combination therapy improved the long‐term survival rate of the mice.

### 3.2 Combination therapy promoted the maturation of MDSCs and induced Th1‐dominant CD4^+^ T‐cell differentiation

**FIGURE 2:**
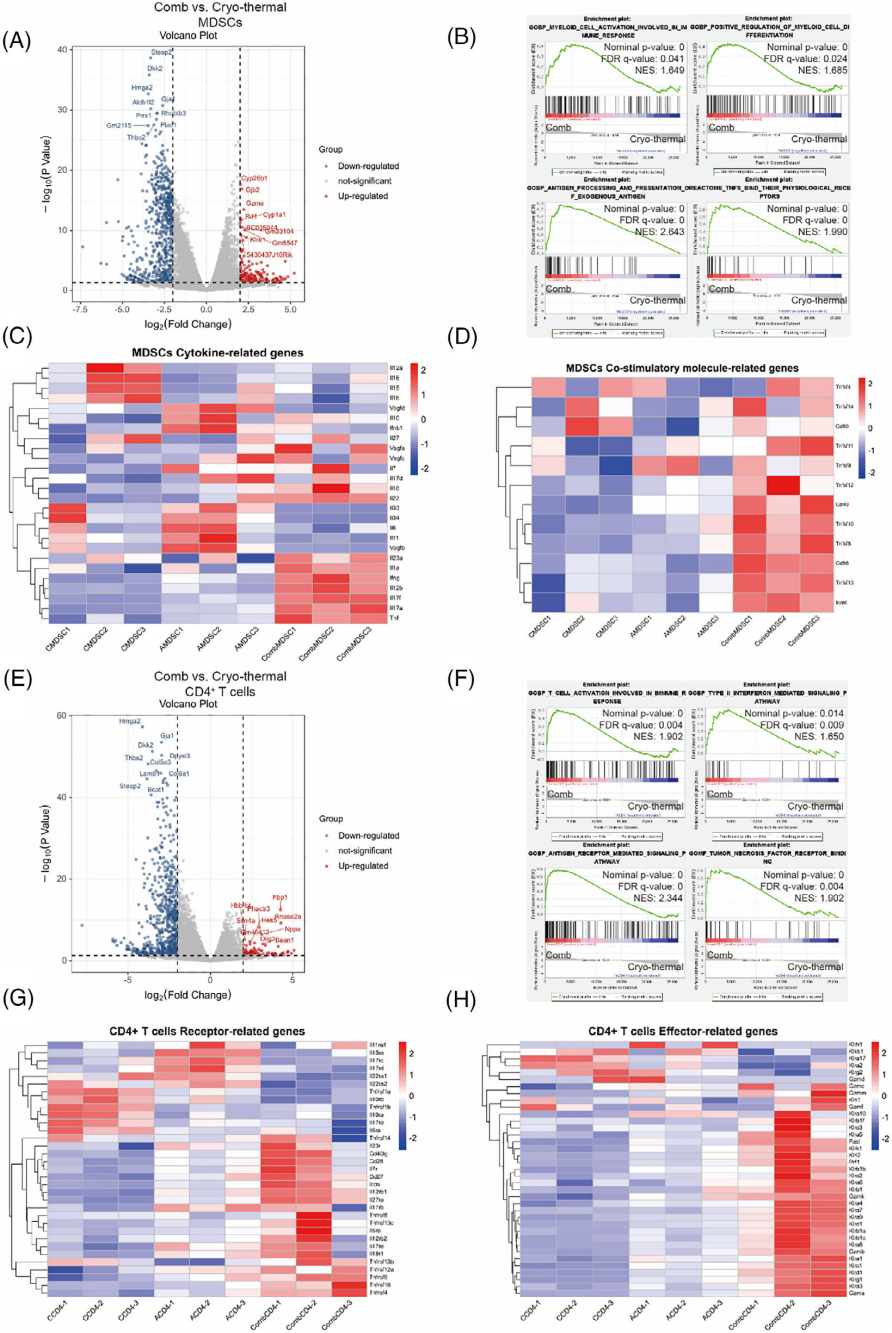
Gene expression profile of MDSCs and CD4^+^ T cells. Pulmonary MDSCs and CD4^+^ T cells from tumour‐bearing group, cryo‐thermal therapy or combination therapy group, and were isolated on Day 14 through Gr‐1^+^ and CD4^+^ magnetic beads separation. The gene expression signature of MDSCs and CD4^+^ T cells were detected by RNA sequence. (A) Volcano map of the different gene expressions of MDSCs (combination therapy compared with cryo‐thermal therapy). (B) Gene set enrichment analysis of myeloid cells activation, myeloid cells differentiation, antigen processing and presentation and TNFs bind their physiological receptors pathways of MDSCs after combination therapy compared with cryo‐thermal therapy. (C) Heatmap of mean fold‐change of cytokine‐related genes expression of MDSCs from tumour‐bearing control (CMDSC), cryo‐thermal therapy (AMDSC), and combination therapy (CombMDSC) group. (D) Heatmap of mean fold‐change of co‐stimulatory molecule‐related genes expression of MDSCs from tumour‐bearing control (CMDSC), cryo‐thermal therapy (AMDSC), and combination therapy (CombMDSC) group. (E) Volcano map of the different gene expressions of CD4^+^ T cells (combination therapy compared with cryo‐thermal therapy). (F) Gene set enrichment analysis of T cell activation involved in immune response, Type II interferon mediated signalling, antigen receptor mediated signalling and TNF receptor binding pathways of CD4^+^ T cells after combination therapy compared with cryo‐thermal therapy. (G) Heatmap of mean fold‐change of receptor‐related genes expression of CD4^+^ T cells from tumour‐bearing control (CCD4), cryo‐thermal therapy (ACD4), and combination therapy (CombCD4) group. (H) Heatmap of mean fold‐change of effector‐related genes expression of CD4^+^ T cells from tumour‐bearing control (CCD4), cryo‐thermal therapy (ACD4), and combination therapy (CombCD4) group. *n* = 3 for each group. MDSCs, myeloid‐derived suppressive cells.

Previous studies have demonstrated that subcutaneous LLC1 tumour model spontaneously develops pulmonary metastatic nodules.^27^, ^28^ Given that CTT achieves complete eradication of primary flank tumours in this model,^21^ we focussed our investigation on lung metastases (the most common site metastasis) to elucidate the mechanisms underlying combination therapy‐induced systemic antitumour immunity. Although the maturity of MDSCs and Th1‐dominant differentiation of CD4^+^ T cells in lungs of LLC1 tumour model have been reported,^22^ which immune cells are directly affected after combination therapy and how they can be reprogrammed to induce strong antitumour immunity remain unclear. We aimed to further understand the role of the neutralization of these two cytokines after CTT in MDSCs maturation and CD4^+^ T‐cell effector function, and performed 3′ RNA sequencing of MACS‐isolated MDSCs and CD4^+^ T cells from the lungs was performed on Day 14 after combination therapy. The results revealed that MDSCs from combination therapy‐treated mice exhibited more robust transcriptional changes than those from CTT‐treated mice (Figure 2A). Hallmark gene set enrichment analysis (GSEA) of MDSCs from mice receiving the combination treatment revealed the significant upregulation of signalling pathways related to maturation and activation, as well as the binding of TNF family receptors and their ligands (Figure 2B). Moreover, an analysis of the expression of cytokine‐related genes in MDSCs revealed that *Tnf* was the most significantly elevated gene (Figure 2C). Heatmap analysis of costimulatory molecule‐related genes revealed that, after combination therapy, multiple members of the TNF superfamily capable of activating T cells through interactions, including *Cd40* (*Tnfrsf5*),^29^ *Tnfrsf10*,^30^ *Tnfrsf8* ^31^ and *Tnfrsf13*,^32^ were activated in MDSCs. Moreover, costimulatory molecules involved in T‐cell activation, including *Cd28* and *Icosl*,^33^ were also significantly activated (Figure 2D). Though most co‐stimulatory molecule‐associated genes were significantly upregulated after combination therapy compared to the tumour‐bearing control and CTT, no significant differences of these genes were observed in the tumour‐bearing control and CTT group. Among these, only *Cd40* was emerged as particularly pattern of change: it was significantly increased after CTT compared to tumour‐bearing control (1.5‐fold, *p* = 0.005) and further elevated after combination therapy (2.2‐fold vs. tumour‐bearing control, *p* < 0.001; 1.47‐fold vs. cryo‐thermal alone, *p* = 0.02). Consequently, a highly distinct pattern of CD40 expression induced after combination therapy promotes our subsequent mechanistic investigation. Meanwhile, RNA‐seq data revealed significant downregulation of metabolic pathways critical for the survival and immunosuppressive function of MDSCs (Figure S2). Additionally, compared with those after CTT, the gene expression signatures of CD4^+^ T cells differed substantially after combination therapy (Figure 2E). The potential antitumour characteristics of CD4^+^ T cells after combination therapy, such as activation, antigen presentation and IFN‐related signalling, were notably revitalized, and the enrichment of TNF receptor binding of TNF on CD4^+^ T cells further indicated crosstalk between MDSCs and CD4^+^ T cells (Figure 2F). As shown in Figure 2G, the expression levels of receptor‐related genes, especially those of TNF superfamily receptor family members, were increased in CD4^+^ T cells after combination therapy. Moreover, the expression of effector functional molecules in CD4^+^ T cells, especially those of the killer cell lectin‐like receptor subfamily and granzyme subfamily, was also detected at the transcriptome level (Figure 2H). These data suggested that the combination therapy could promote MDSCs maturation and stimulate CD4^+^ T‐cell‐mediated antitumour immunity. Interestingly, the upregulation of TNF family member receptor and ligand genes in MDSCs and CD4^+^ T cells indicated a means of communication between MDSCs and CD4^+^ T cells.

**FIGURE 3:**
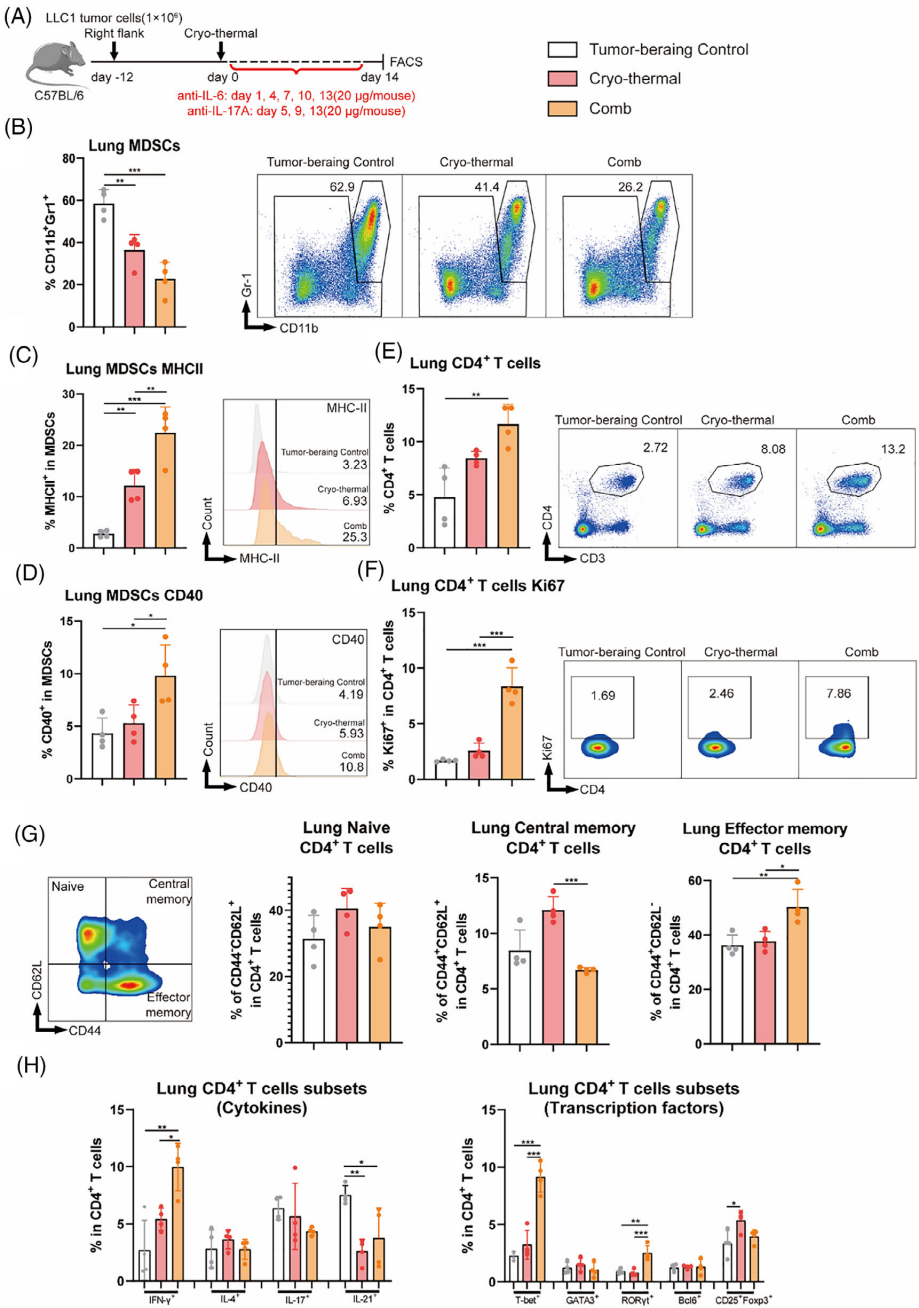
Combination therapy promoted the maturation of MDSCs and induced Th1‐dominant CD4^+^ T‐cell differentiation. (A) Scheme of study design. In brief, LLC1 tumour‐bearing mice were intraperitoneally treated with PBS or 20 µg of anti‐IL‐6 and IL‐17A, the phenotype of MDSCs and the subsets of CD4^+^ T cells were detected on Day 14 by flow cytometry. (B–D) The proportion (B), MHCII (C), and CD40 (D) of MDSCs. (E–H) The proportion (E), proliferation (F), phenotype (G), and subset (H) of CD4^+^ T cells. *p <0.05, **p<0.01, ***p <0.001. n = 4 for each group.

To further determine the effects of IL‐6 and IL‐17A neutralization after CTT on the immune function of pulmonary MDSCs and CD4^+^ T cells in vivo (Figure 3A). The proportions, phenotypes and functions of MDSCs and CD4^+^ T cells were analysed on day 14 after CTT using flow cytometry. As shown in Figure 3B, the combination therapy further inhibited the accumulation of MDSCs in the lungs compared with that after CTT. We investigated whether the combination therapy could facilitate the maturation and differentiation of MDSCs by further analysing the expression levels of surface markers, including CD86, MHCII, CD40, CD11c and F4/80, on MDSCs. The proportion of MHCII^+^ MDSCs, which are mature MDSCs,^31^ was markedly greater after combination therapy than after CTT, and the proportion of MHCII^+^ MDSCs after CTT was also significantly greater than that of MDSCs from tumour‐bearing controls (Figure 3C). Compared with those in the other two groups, the expression levels of CD40 on MDSCs were notably increased after the combination therapy (Figure 3D). The proportions of PMN‐MDSCs and M‐MDSCs and the expression levels of MHCII and CD40 were consistent among them (Figure S3A–F), and subsequent investigations analysed MDSCs as a unified population without further subcategorization. However, the expression levels of CD86 and CD11c on MDSCs were not significantly different after combination therapy and CTT, and the expression of F4/80 on MDSCs decreased after combination therapy (Figure S3G–I).

Moreover, the proportion of pulmonary CD4^+^ T cells sharply increased after combination therapy compared with CTT (Figure 3E). Consistently, the proliferation of CD4^+^ T cells after combination therapy, as indicated by Ki67^+^ labelled cells,^34^ was markedly higher than that in the tumour‐bearing control and cryo‐thermal groups (Figure 3F). In addition, the proportion of central memory (CD44^+^CD62L^+^) CD4^+^ T cells was lower after combination therapy than after CTT (Figure 3G). Moreover, a higher frequency of effector memory (CD44^+^CD62L^−^) CD4^+^ T cells was observed after combination therapy than after CTT alone (Figure 3G). Interestingly, the proportion of the Th1 subset (IFN‐γ^+^ and T‐bet^+^) was notably increased only after combination therapy, which indicated that Th1 cell differentiation was predominant in the lung (Figure 3H). Collectively, these findings indicated that, compared with CTT, combination therapy inhibited the accumulation of MDSCs and facilitated their maturation, as indicated by the high expression levels of CD40. Moreover, the expansion, effector phenotype and Th1‐dominant differentiation of CD4^+^ T cells were markedly induced in the lungs after combination therapy.

### 3.3 IL‐6 and IL‐17A neutralization directly promoted the expression levels of CD40 and TNF‐α on pulmonary MDSCs after cryo‐thermal therapy

**FIGURE 4:**
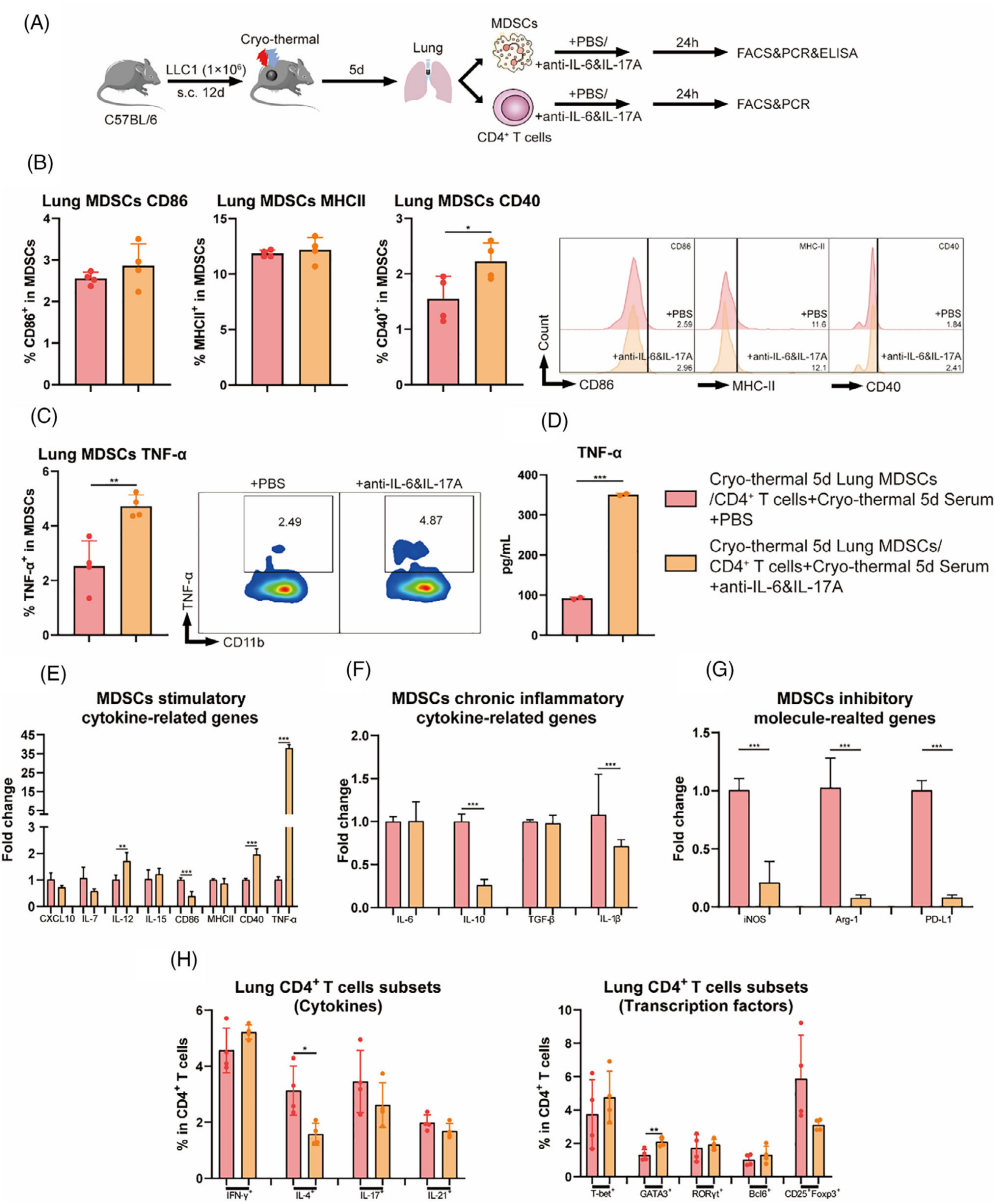
In vitro the phenotype of MDSCs and the subsets of CD4^+^ T cells after cryo‐thermal therapy with IL‐6 and IL‐17A neutralization. (A) Scheme of study design. In brief, MDSCs and CD4^+^ T cells after cryo‐thermal therapy on day 5 were separated and co‐cultured with or without IL‐6 and IL‐17A neutralization (10 ng/mL). The corresponding serums were added to mimic the cytokine environment in vivo. The phenotype of MDSCs and the subsets of CD4^+^ T cells were detected after 24 h by flow cytometry. (B) The maturation phenotype of MDSCs (*n* = 4). (C) The expression levels of TNF‐α in MDSCs were detected by flow cytometry (*n* = 4). (D) The concentration of TNF‐α in culture supernatants were detected by ELISA (*n* = 2). (E–G) The molecule expression profiles of pulmonary MDSCs were detected by qRT‐PCR (*n* = 4). (H) The subsets of CD4^+^ T cells. *p <0.05, **p<0.01, ***p <0.001.

The LLC1 subcutaneous tumour model develops spontaneous lung metastases and the flank tumours were completely eradicated by CTT. Therefore, to further investigate which cells were directly affected by the neutralization of these two cytokines after CTT and the corresponding mechanisms of systemic anti‐tumour immunity against lung metastases after combination therapy, pulmonary MDSCs or CD4^+^ T cells on day 5 (the first time point of in vivo injection of IL‐17A neutralizing antibody) after CTT were isolated via magnetic‐activated cell sorting (MACS) and cultured in vitro for 24 h with or without the addition of IL‐6 and IL‐17A neutralizing antibodies and with 10% of the corresponding mice serum to mimic the in vivo environment (Figure 4A). Compared with those in the CTT alone group, no significant changes in the levels of CD86 and MHCII on MDSCs were observed with the addition of neutralizing antibodies (Figure 4B). Unexpectedly, the expression levels of CD40 on MDSCs after CTT with neutralizing IL‐6 and IL‐17A antibodies were significantly increased (Figure 4B). Moreover, higher expression levels of TNF‐α in MDSCs and higher concentrations of TNF‐α in the corresponding culture supernatants were observed after CTT with neutralizing IL‐6 and IL‐17A antibodies (Figure 4C,D). The expression levels of functional molecules in MDSCs were detected via qRT‒PCR to further analyse the characteristics of MDSCs after CTT with in vitro cytokine neutralization. As shown in Figure 4E, compared with the levels detected in MDSCs after CTT, in vitro cytokine neutralization dramatically upregulated the expression of stimulatory molecules, including IL‐12, CD40 and especially TNF‐α. The expression of IL‐10, iNOS, Arg‐1 and PD‐L1 in MDSCs was markedly downregulated (Figure 4F,G). However, CD4^+^ T cells did not differentiate towards the Th1 subtype in vitro following cytokine neutralization, which was inconsistent with the in vivo results (Figure 4H). These results suggested that cytokine neutralization directly affected MDSCs and promoted the expression of CD40 and TNF‐α after CTT, whereas the effect of cytokine neutralization on CD4^+^ T‐cell differentiation was indirect.

### 3.4 Pulmonary MDSCs isolated after combination therapy facilitated Th1‐dominant CD4+ T‐cell differentiation via the TNF‐α‒CD40/CD40L axis

**FIGURE 5:**
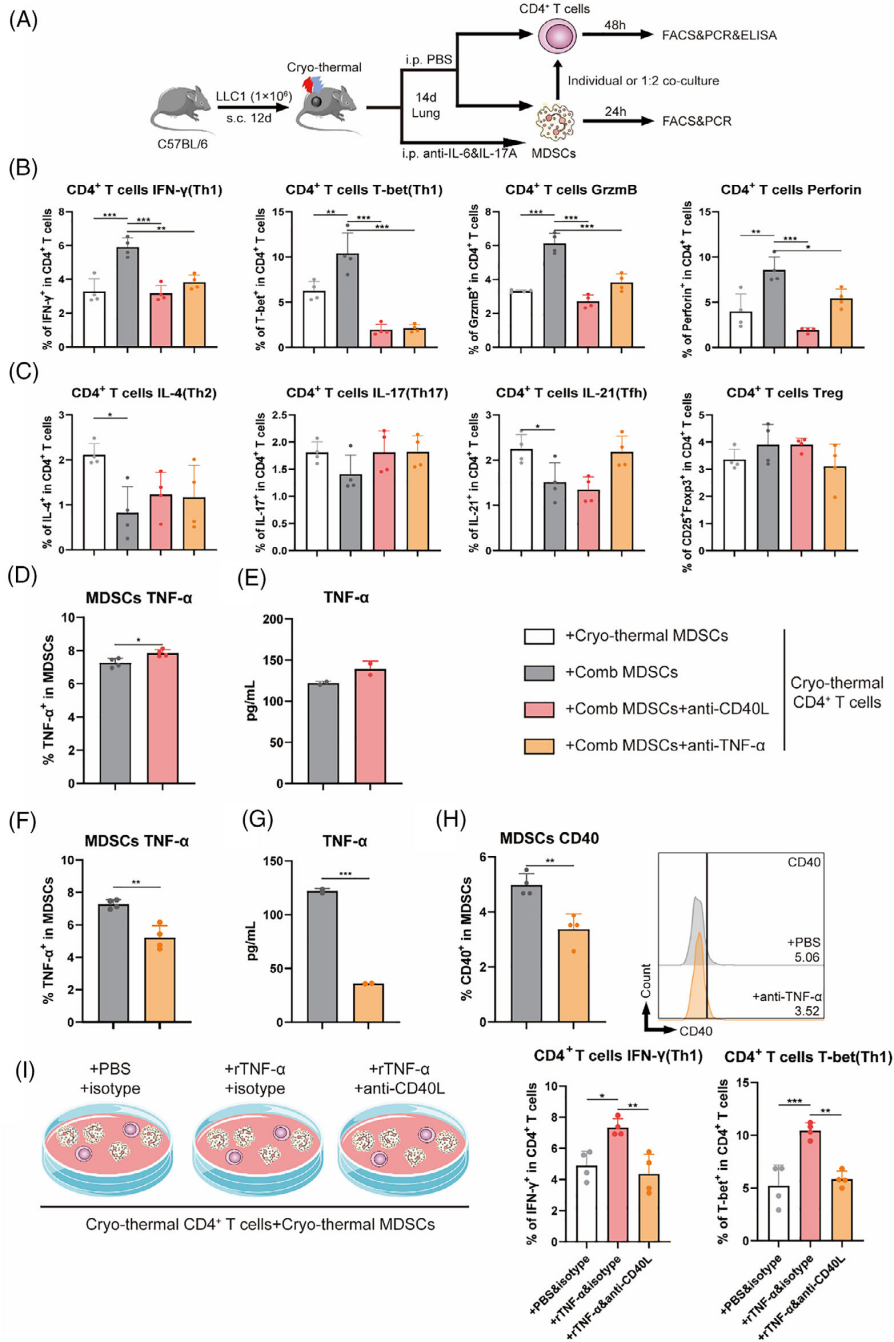
Pulmonary MDSCs after combination therapy facilitated Th1‐dominant CD4^+^ T cell differentiation via the TNF‐α and CD40‐CD40L axis. (A) Scheme of study design. In brief, CD4^+^ T cells after cryo‐thermal therapy on Day 14 were separated and co‐cultured with MDSCs after cryo‐thermal or combination therapy in the ratio of 1:2 with or without CD40L blockade (10 ng/mL) and TNF‐α monoclonal antibody (10 ng/mL), respectively. The corresponding serums were added to mimic the cytokine environment in vivo. (B, C) The subsets of CD4^+^ T cells co‐cultured with MDSCs. (D) The expression levels of TNF‐α in MDSCs were detected by flow cytometry (n = 4). (E) The concentration of TNF‐α in culture supernatants were detected by ELISA (n = 2). (F) The expression levels of TNF‐α in MDSCs were detected by flow cytometry (n = 4). (G) The concentration of TNF‐α in culture supernatants were detected by ELISA (n = 2). (H) The expression levels of CD40 on MDSCs (*n* = 4). (I) The subsets of CD4^+^ T cells co‐cultured with MDSCs addition of PBS + isotype, recombinant TNF‐α (10 ng/mL)+isotype, and recombinant TNF‐α (10 ng/mL) + anti‐CD40L, respectively. *p <0.05, **p<0.01, ***p <0.001. *n* = 4 for each group.

The combination therapy directly increased the expression levels of TNF‐α and CD40 in MDSCs, which have been reported to promote the Th1‐dominant differentiation of CD4^+^ T cells in acute infections and autoimmune diseases.^35^, ^36^ Moreover, at the genetic level, the upregulation of TNF family member receptors and ligands in MDSCs and CD4^+^ T cells was observed via RNA‐seq. We hypothesized that after combination therapy, mature MDSCs would promote Th1‐dominant differentiation, possibly via the CD40/CD40L interaction or TNF‐α. To explore how MDSCs directly regulate CD4^+^ T cell differentiation after combination therapy, CD4^+^ T cells and MDSCs from CTT or combination therapy group were isolated on day 14 after treatment (the timepoint when we observed the maturation of MDSCs and Th1‐dominant CD4^+^ T cell differentiation in vivo) with or without CD40L blockade and treatment with a TNF‐α monoclonal antibody, respectively. The schematic of the study design is shown in Figure 5A. Compared with those in co‐cultures with MDSCs isolated after CTT, CD4^+^ T cells in co‐cultures with MDSCs isolated after combination therapy exhibited Th1 differentiation (as evidenced by the expression levels of the cytokine IFN‐γ and the transcription factor T‐bet) and increased effector function (as evidenced by the expression levels of granzyme B and perforin) (Figure 5B). However, these increases were abrogated by the blockade of CD40L or neutralization of TNF‐α (Figure 5B), which suggested that after the combination therapy, MDSCs were able to promote CD4^+^ T‐cell differentiation towards the Th1 subset and increase their effector function via the CD40/CD40L interaction or TNF‐α. As shown in Figure 5C, compared with cells in co‐cultures with MDSCs isolated after CTT, the proportions of Th2 and Tfh cells among CD4^+^ T cells were decreased in co‐cultures with MDSCs isolated after the combination therapy. In addition, no differences in the proportions of Th17 and Treg cells were observed between the groups (Figure 5C). CD4^+^ Th1 cells are characterized by high expression of two cytokines, IFN‐γ and TNF‐α.^37^ MACS‐isolated CD4^+^ T cells after CTT were cultured in vitro with or without a TNF‐α monoclonal antibody. In addition, different concentrations of recombinant TNF‐α were added to the cell culture medium of MACS‐isolated CD4^+^ T cells after CTT. Neither the addition nor the neutralization of TNF‐α had a direct effect on the differentiation of CD4^+^ T cells (Figure S4). In summary, the induction of CD4^+^ Th1 differentiation in MDSCs after combination therapy was dependent on the indirect effect of TNF‐α production and the direct effect of CD40 on the cell surface.

MACS‐isolated CD4^+^ T cells after CTT were co‐cultured in vitro with MDSCs isolated after combination therapy with or without treatment with CD40L blockade/TNF‐α monoclonal antibodies to investigate whether high expression levels of CD40 and TNF‐α in MDSCs induced by combination therapy can affect each other. As shown in Figure 5D,E, blockade of the CD40*/*CD40L interaction did not decrease the expression levels of TNF‐α or the concentration of TNF‐α in cell culture supernatants from MDSCs isolated after combination therapy. However, TNF‐α neutralization decreased the expression levels of TNF‐α in MDSCs and the concentration of TNF‐α in cell culture supernatants from MDSCs isolated after combination therapy (Figure 5F,G). Moreover, TNF‐α neutralization significantly reduced the expression levels of CD40 on MDSCs isolated after combination therapy (Figure 5H). These results suggested that, after combination therapy, MDSCs promoted the expression of CD40 through the production of TNF‐α.

We validated whether the induction of Th1 differentiation via TNF‐α in MDSCs isolated after combination therapy relied on the regulation of CD40 by co‐culturing MACS‐isolated CD4^+^ T cells after CTT with MDSCs isolated after CTT with the addition of recombinant TNF‐α and blockade of both recombinant TNF‐α and CD40L. Compared with cells co‐cultured with MDSCs isolated after CTT, CD4^+^ T cells co‐cultured with MDSCs isolated after CTT with the addition of recombinant TNF‐α underwent Th1 cell differentiation (as evidenced by the expression levels of the cytokine IFN‐γ and the transcription factor T‐bet) (Figure 5I). However, this change was abrogated after the blockade of CD40L (Figure 5I). Moreover, Th1‐dominant CD4^+^ T cells after combination therapy, which enhanced the effector function of CD8^+^ T cells compared with CD4^+^ T cells from the tumour‐bearing and CTT group. (Figure S5A–C). These data indicated that combination therapy promoted the production of TNF‐α in MDSCs, which induced the expression of CD40 on MDSCs to trigger the Th1‐dominant differentiation of CD4^+^ T cells via the CD40/CD40L interaction.

### 3.5 Mature pulmonary MDSCs isolated after combination therapy promote Th1 differentiation through CD40 induced by autocrine TNF‐α via the NF‐κB signalling pathway

**FIGURE 6:**
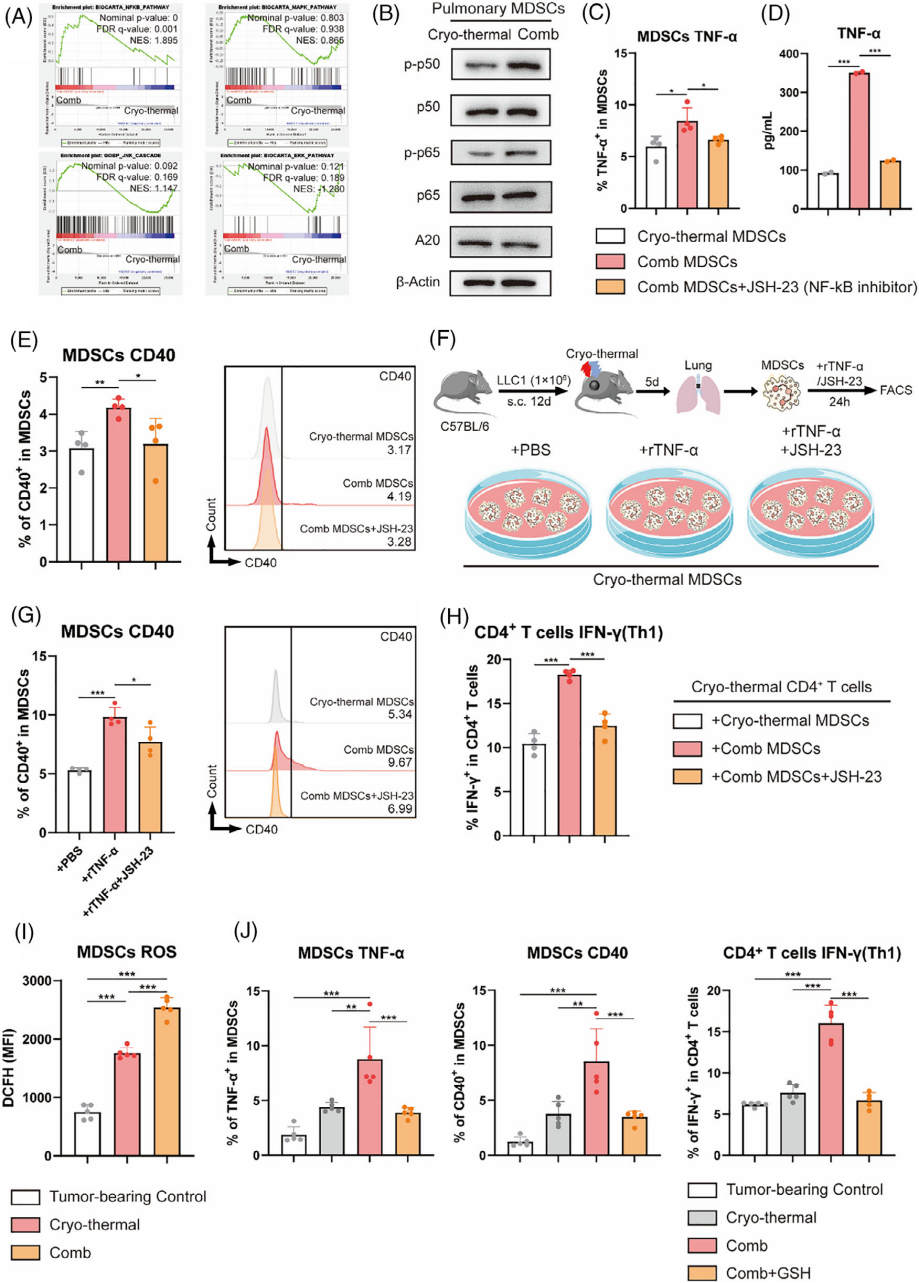
Pulmonary mature MDSCs after combination therapy elevated autocrine production of TNF‐α and the expression levels of CD40 and through enhanced activation of NF‐κB signalling pathway. (A) Gene set enrichment analysis of NF‐κB, MAPK, JNK and ERK pathway of MDSCs after combination therapy compared with cryo‐thermal therapy. (B) The levels of NF‐κB p‐p50, NF‐κB p50, NF‐κB p‐p65, NF‐κB p65 and A20 in MDSCs after cryo‐thermal or combination were detected by western blot (n = 3). (C) The expression levels of TNF‐α in MDSCs with or without treatment with JSH‐23 (10 µM) were detected by flow cytometry (*n* = 4). (D) The concentration of TNF‐α in culture supernatants were detected by ELISA (*n* = 2). (E) The expression levels of CD40 on MDSCs (*n* = 4). (F) Scheme of study design. In brief, MDSCs after cryo‐thermal therapy on Day 5 were separated and co‐cultured addition of recombinant TNF‐α (10 ng/mL), and both recombinant TNF‐α (10 ng/mL) and JSH‐23 (10 µM), respectively. (G) The expression levels of CD40 on MDSCs were detected after 24 h by flow cytometry (*n* = 4). (H) The Th1 subset of CD4^+^ T cells co‐cultured with MDSCs isolated after cryo‐thermal therapy or combination therapy with or without the addition of JSH‐23 (10 µM) (n = 4). (I) The MFI of ROS in MDSCs on day 14 after cryo‐thermal therapy (n = 5). (J) The expression levels of TNF‐α and CD40 on MDSCs and IFN‐γ in CD4^+^ T cells were detected by flow cytometry (*n* = 5). *p <0.05, **p<0.01, ***p <0.001.

Activation of the NF‐κB, MAPK, JNK and ERK pathways can promote TNF‐α production in neuroglia, mesenchymal stem cells and tumour cells.^38^, ^39^, ^40^, ^41^ As described previously, MDSCs were isolated via MACS after CTT and combination therapy, and 3′ RNA sequencing was performed to explore the mechanisms underlying the modulation of TNF‐α production in MDSCs after combination therapy. The above signalling pathways were analysed via GSEA, which revealed that only the positive regulation of NF‐κB signalling was significantly enriched after combination therapy compared with that after CTT, and no differences in other pathways were observed (Figure 6A). The level of the phosphorylated NF‐κB‐p50, NF‐κB‐p65 subunits and A20 in MDSCs after combination therapy was examined using western blotting (Figure 6B). The results revealed that the levels of p‐p50/p50 and p‐p65/p65 were significantly higher in MDSCs after combination therapy than in MDSCs after CTT, while the levels of A20 in MDSCs were significantly decreased after combination therapy, indicating the activation of the NF‐κB pathway in MDSCs after the combination therapy. TNF‐α is a key upstream cytokine that activates the NF‐κB pathway,^42^, ^43^ and we speculated that the high expression levels of TNF‐α detected in MDSCs after combination therapy promoted self‐expression through the NF‐κB pathway. After cultured in vitro MDSCs after combination therapy with addition of the NF‐κB inhibitor JSH‐23 to cultured MDSCs in vitro, the high expression levels of TNF‐α detected in MDSCs after combination therapy were markedly decreased (Figure 6C). Consistently, the concentration of TNF‐α in the culture supernatants was significantly decreased (Figure 6D). These data indicated that combination therapy activated NF‐κB signalling in MDSCs, thereby inducing TNF‐α production.

MDSCs from the treated mice on day 14 after CTT or combination therapy were isolated via MACS and cultured in vitro to investigate whether TNF‐α produced by MDSCs after combination therapy can promote the expression of CD40 via NF‐κB signalling. The expression levels of CD40 on pulmonary MDSCs were markedly increased after combination therapy, but the increased expression levels of CMDSCs after combination therapy were abolished by the addition of JSH‐23 (Figure 6E). To investigate the mechanisms underlying the increased protein and gene levels of TNF‐α in MDSCs induced by neutralizing cytokines, pulmonary MDSCs obtained from the treated mice on day 5 (the time point at which cytokines neutralization in vitro directly induces the upregulation of TNF‐α) after CTT were isolated via MACS and cultured in vitro for 24 h with the addition of recombinant TNF‐α or both recombinant TNF‐α and JSH‐23 to verify whether TNF‐α signalling via the NF‐κB pathway affects the maturation of MDSCs after combination therapy (Figure 6F). The expression levels of CD40 on MDSCs were markedly increased upon the addition of recombinant TNF‐α. This change was consistently reversed by the inhibition of NF‐κB signalling (Figure 6G). CD4^+^ T cells isolated after CTT were co‐cultured with MDSCs isolated after CTT or combination therapy with or without the addition of JSH‐23 to explore whether the reduction in the levels of TNF‐α in MDSCs induced by NF‐κB inhibition further affects the Th1‐dominant differentiation of CD4^+^ T cells. Compared with cells co‐cultured with MDSCs isolated after CTT, CD4^+^ T cells co‐cultured with MDSCs isolated after the combination therapy exhibited Th1 differentiation (IFN‐γ^+^ and T‐bet^+^ proportions), but the inhibition of the NF‐κB pathway reversed the induction of Th1 polarization in CD4^+^ T cells (Figure 6h).

Reactive oxygen species (ROS) are established activators of the NF‐κB signalling pathway through their ability to modify key regulatory proteins.^41^ Our data demonstrate that CTT significantly enhances both ROS biosynthesis and NF‐κB pathway activity in MDSCs relative to tumour‐bearing controls (Figure S6A,B). This effect was further amplified when CTT was combined with IL‐6/IL‐17A neutralization, which produced additional augmentation of ROS biosynthetic pathways in MDSCs (Figure S6C). Flow cytometric analysis on Day 14 post‐treatment confirmed these findings, revealing substantially elevated ROS levels in pulmonary MDSCs after CTT, with combination therapy yielding even greater ROS induction (Figure 6I). Therefore, we administered GSH intraperitoneally to mice in the combination therapy group to eliminate ROS in vivo. We found that the expression of CD40 and TNF‐α in MDSCs was significantly reduced after ROS elimination compared to combination therapy alone, and the proportion of Th1 in CD4^+^ T cells also decreased significantly after ROS elimination (Figure 6J). These findings demonstrate that IL‐6 and IL‐17A neutralization after CTT induces ROS‐mediated NF‐κB pathway activation in pulmonary MDSCs and subsequent autocrine TNF‐α production, which further activated the NF‐κB signalling pathway and led to high expression of CD40 on MDSCs, thereby promoting the Th1‐dominant differentiation in CD4^+^ T cells.

### 3.6 Combination therapy promoted the maturation of MDSCs and induced Th1‐dominant CD4^+^ T‐cell differentiation of distal tumours in the MC38 bilateral tumour model

**FIGURE 7:**
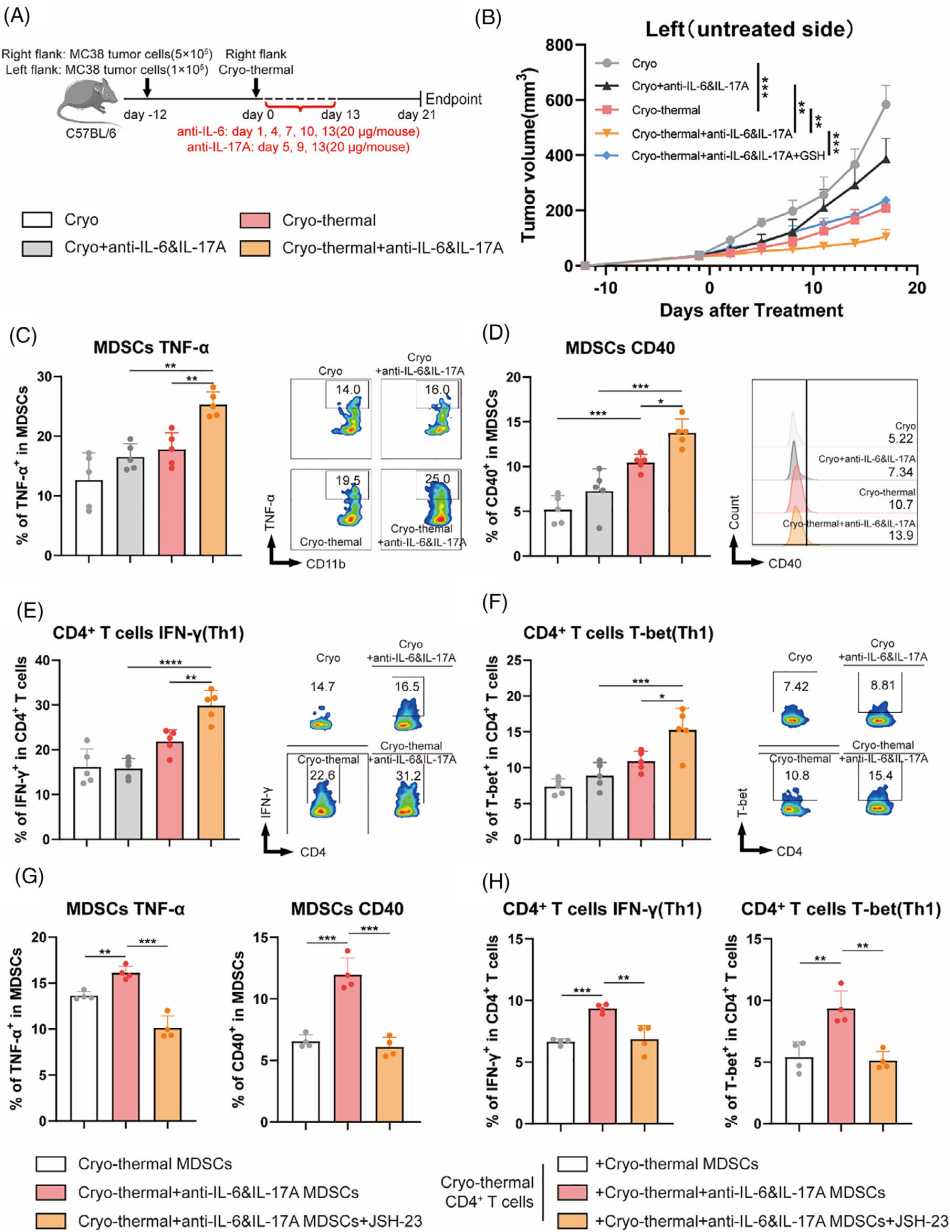
Combination therapy promoted the maturation of MDSCs and induced Th1‐dominant CD4^+^ T‐cell differentiation of distal tumours in the MC38 bilateral tumour model. (A) Scheme of study design. In brief, MC38 tumour‐bearing mice (*n* = 5/group) were performed cryo‐thermal therapy on only one side (cryoablation as control) and intraperitoneally treated with PBS or 20 µg of anti‐IL‐6 and IL‐17A or 10 mg/kg GSH. (B) The untreated side tumour growth of mice was monitored. The tumour growth curves were compared using two‐way ANOVA (n = 5/group). (C) The expression levels of TNF‐α in MDSCs (n = 5). (E) The expression levels of IFN‐γ in CD4^+^ T cells (*n* = 5). (D) The expression levels of CD40 on MDSCs (*n* = 5). (F) The expression levels of T‐bet in CD4^+^ T cells (*n* = 5). (G) The expression levels of TNF‐α and CD40 on MDSCs with or without treatment with JSH‐23 (10 µM) were detected by flow cytometry (*n* = 4). (H) The Th1 subset of CD4^+^ T cells co‐cultured with MDSCs isolated after cryo‐thermal therapy or combination therapy with or without the addition of JSH‐23 (10 µM) *(n* = 4). *p <0.05, **p<0.01, ***p <0.001. *n* = 5 for each group.

To visually abscopal effect induced by combination therapy, MC38 subcutaneous bilateral tumour models was used to treated one side of tumours by using CTT and monitor the growth of the untreated side tumours (*n* = 5 for each group, Figure 7A). Cryoablation, which has demonstrated clinical efficacy in inducing tumour‐specific immune responses,^42^ served as the control treatment. Notably, CTT combined with IL‐6 and IL‐17A neutralization was demonstrated to remarkable inhibition of distal tumour growth compared with both CTT alone and cryoablation combined with IL‐6 and IL‐17A neutralization (Figure 7B). At the endpoint, the untreated side tumours in each group were photographed (Figure S7).

In LLC1‐bearing mice, we comprehensively explored the mechanism of CD40 induced by TNF‐α produced in an autocrine manner on reprogrammed MDSCs in lungs (as potential metastatic site) after combination therapy (as shown in Figure 1, 2, 3, 4, 5, 6). To further validate the mechanism, we constructed a bilateral tumour model using MC38 cells and performed CTT on only one side to verified whether combination therapy have the same effect on MDSCs in distal untreated tumours. At experimental endpoint, we assessed immunological alterations in MDSCs and CD4^+^ T cells within the untreated contralateral tumours using flow cytometry. CTT significantly increased the expression of CD40 on MDSCs compared with cryoablation, which was further enhanced after combination therapy (Figure 7C). The level of TNF‐α in MDSCs did not significantly differ in cryoablation and CTT groups, while the levels of TNF‐α in MDSCs was markedly elevated after combination therapy compared with cryoablation combined with IL‐6 and IL‐17A neutralization and cryo‐thermal alone (Figure 7D). The proportion of Th1 subset (IFN‐γ^+^ and T‐bet^+^) cells presented a significant increase exclusively after combination therapy (Figure 7E,F), consistent with observations in the LLC1 model.

To validate the regulatory role of the NF‐κB signalling pathway in MDSCs after combination therapy in the MC38 model, MDSCs from the untreated side tumours were sorted on day 14 after CTT or combination therapy. After being cultured in vitro MDSCs after combination therapy with addition of JSH‐23 to cultured MDSCs in vitro, the high expression levels of TNF‐α and CD40 (compared to CTT) detected on MDSCs after combination therapy were markedly decreased (Figure 7G). CD4^+^ T cells isolated on day 14 after CTT were co‐cultured with MDSCs isolated on Day 14 after CTT or combination therapy with or without the addition of JSH‐23 to explore whether the NF‐κB pathway affects the Th1‐dominant differentiation of CD4^+^ T cells in the MC38 model. Compared with cells co‐cultured with MDSCs isolated after CTT, CD4^+^ T cells co‐cultured with MDSCs isolated after the combination therapy exhibited Th1 differentiation (IFN‐γ^+^ and T‐bet^+^ proportions), but the inhibition of the NF‐κB pathway reversed the induction of Th1 polarization in CD4^+^ T cells (Figure 7H). The above results indicated that activation of the NF‐κB signalling pathway in MDSCs in the untreated side tumours of the MC38 model upregulates the expression of TNF‐α and CD40, thereby inducing Th1‐dominant CD4^+^ T cell differentiation, consistent with the mechanism in the lungs of LLC1 model (Figure 6). Collectively, these findings demonstrate that combination therapy enhances the expression of CD40 and TNF‐α in MDSCs while promoting Th1‐dominant CD4^+^ T cell differentiation in distal untreated tumours within the MC38 bilateral tumour model, thereby effectively suppressing distal tumour growth.

## 4 DISCUSSION

In previous study, we reported that combining CTT with IL‐6 and IL‐17A neutralization induced the maturation of MDSCs and the Th1‐dominant differentiation of CD4^+^ T cells, thereby remodelling the tumour immune microenvironment in LLC1 lung cancer and leading to long‐term antitumour immunity.^22^ However, the mechanisms underlying the direct effects of CTT combined with IL‐6 and IL‐17A neutralization on MDSCs and CD4^+^ T cells and how MDSCS maturation and the Th1 differentiation of CD4^+^ T cells are induced after combination therapy are still unknown. In this study, we found that the combination therapy markedly induced the production of TNF‐α in MDSCs via the activation of NF‐κB signalling, which further increased the level of CD40 expression on MDSCs. Mature MDSCs with high expression levels of CD40 effectively promoted the Th1 differentiation of CD4^+^ T cells via the CD40–CD40L interaction, resulting in the long‐term survival of these mice.

IL‐6 and IL‐17A can promote lung cancer progression by regulating several signalling mechanisms.^43^ The production of IL‐17A in the lung amplifies the autophagy‐activating effect of IL‐6 by activating the TRAF6 pathway in monocytes, M2 macrophages (TAMs) and type III immune cells (including tumour‐associated fibroblasts and endothelial cells), which leads to immune escape.^44^ Given the ability of IL‐6 and IL‐17A to maintain lung tumour cell survival and increase susceptibility to lung cancer, a growing number of studies have confirmed that high levels of IL‐6 and IL‐17A can be used to predict a poor prognosis for lung cancer patients. Moreover, a cross‐sectional study comparing the plasma IL‐6 and IL‐17 levels between lung cancer patients and healthy individuals revealed that these two cytokines can be used as diagnostic indicators of lung cancer.^45^ However, to date, monoclonal antibodies against IL‐6 and IL‐17A have not been used in preclinical and clinical studies of lung cancer treatment. In this study, therapeutic targeting of IL‐6 and IL‐17A with neutralizing antibodies only prolonged the survival time of the mice but did not improve the survival rate of the mice compared with that of the tumour‐bearing controls, possibly because the immunosuppressive environment induced by the tumour load in situ was difficult to reverse. The ability of CTT alone to prolong the long‐term survival of LLC1‐bearing mice may be due to the inability to fully reverse the immunosuppressive environment. However, CTT combined with IL‐6 and IL‐17A neutralization not only completely ablated tumours in situ but also effectively eliminated the immunosuppressive microenvironment, leading to improved long‐term survival (50%).

MDSCs, which have recently emerged as a major cell population, are regulators of immune responses in the tumour microenvironment, where they promote tumour growth by exerting their immunosuppressive functions. Moreover, MDSCs have always been key therapeutic targets in the treatment of cancer. However, MDSCs, as immature myeloid cells, were shown to have functional plasticity in our previous studies.^46^ In B16F10 melanoma‐bearing mice, natural killer cells reprogram MDSCs to induce TNF‐α release, which promotes the maturation of MDSCs after CTT.^47^ In this study, CTT alone only prolonged the long‐term survival of LLC1 mice, possibly because of the inability of CTT alone to induce the release of high levels of TNF‐α in MDSCs and thereby promote their maturation. Currently, the mechanism by which CD40 expression is induced by TNF‐α via the NF‐κB pathway has been reported for endothelial cells and renal inner medullary collecting duct cells.^38^, ^39^ However, the related mechanism in MDSCs has not yet been investigated.

CTT is an innovative immunotherapeutic approach that combines pre‐freezing, thermotherapy and thermophysical induction. This technique first pretreated tumour tissue by cryoablation, followed treatment by radiofrequency heating. The dramatic temperature fluctuations and mechanical stress induced by this process disrupt tumour cells in situ and damage the tumour microcirculation leading to the abundant release of immune‐stimulating molecules and tumour‐associated antigens, which effectively reverses systemic immunosuppression, creating a favourable microenvironment for enhancing systemic anti‐tumour immune response to eliminate cancer cells.^20^, ^48^ Moreover, the efficient presentation of tumour antigens on APCs induced by CTT stimulates the generation, activation and proliferation of tumour‐specific T cells to significantly elevate the population of circulating and tissue‐resident tumour‐specific T cells, thereby triggering a robust systemic anti‐tumour immunity.^21^ Because simultaneously enabling local tumour control and inducing systemic therapeutic effects, CTT not only effectively treats primary solid tumours, but also inhibits recurrence and metastasis, offering a comprehensive tumour treatment strategy. These effects underlie the significant improvement in long‐term survival observed in the LLC1 model after CTT. Notably, CTT elevated serum levels of both IL‐6 and IL‐17A, suggesting these cytokines may serve as targets for combination therapy to further improve therapeutic efficacy in the LLC1 model.^48^ This study specifically investigated the immune mechanisms underlying the enhanced antitumour efficacy of CTT combined with IL‐6/IL‐17A neutralization. Our results showed that combination therapy promoted the maturation of MDSCs expressing CD40 via high levels of TNF‐α production caused by the activation of the NF‐κB pathway. Our study revealed that the combination of IL‐6 and IL‐17A neutralizing antibodies and CTT promotes the expression of CD40 on MDSCs by inducing TNF‐α production via the NF‐κB pathway in MDSCs. The CD40–CD40L interaction initiates the Th1‐dominant differentiation of CD4^+^ T cells, leading to long‐term antitumour immunity. This study highlights the mechanism of CD40 regulation by TNF‐α produced in an autocrine manner by reprogrammed MDSCs after immunotherapy.

In this study, we validated the efficacy of the combination therapy in preclinical animal models. CTT ablation system has been approved by the National Medical Products Administration for the treatment of clinical primary and metastatic liver cancer and lung cancer (20233010773). In lung tumour treatment, traditional cryoablation is often limited by prolonged procedure times due to haemorrhage risks, and radiofrequency ablation faces challenges in precise range control. However, by using ultra‐micro‐probes and precise thermal dosage regulation, CTT reduces haemorrhage, minimizes pain, lowers complication rates and facilitates faster recovery. In addition, currently approved IL‐6 and IL‐17A monoclonal antibody drugs are already in clinical use for the treatment of diseases such as rheumatoid arthritis and psoriasis. In clinical studies for tumour treatment, siltuximab (anti‐IL‐6 monoclonal antibody) delay the progression of multiple myeloma in clinical trial.^47^ Secukinumab (anti‐IL‐17 monoclonal antibody) combined with Camrelizumab (anti‐PD‐1 monoclonal antibody) effectively inhibited in situ and distal tumour growth in patients with advanced carcinoma of colon.^49^, ^50^

Considering timing optimization of clinical protocols for combination therapy, we will refer to the dosing strategy in preclinical animal models and perform dynamic monitoring of serum IL‐6 and IL‐17A levels in patients after CTT. Given the propensity of IL‐6 monoclonal antibodies to induce upper respiratory tract infections and elevated liver enzymes, low doses of antibodies used reduces the risk, meanwhile, regular monitoring for influenza, pneumococcal infections and liver function tests are warranted throughout the combination therapy process.^51^ While IL‐17A monoclonal antibodies demonstrate a more favourable safety profile overall, their use should be avoided in patients with active inflammatory bowel disease.^52^ Therefore, CTT combined with IL‐6 and IL‐17A monoclonal antibody would be a highly promising treatment strategy for patients with non‐small cell lung cancer and other types of cancer.

## AUTHOR CONTRIBUTIONS

**Lisa X. Xu** and **Ping Liu**: Conceptualization. **Lisa X. Xu** and **Ping Liu**: Funding acquisition. **Yuankai Hao, Shicheng Wang, Junjun Wang, Zelu Zhang, Yichen Yao** and **Ke Wang**: Investigation. **Yuankai Hao**: Methodology. **Ping Liu**: Project administration. **Lisa X. Xu** and **Ping Liu**: Supervision. **Yuankai Hao**: Visualization. **Yuankai Hao**: Writing–original draft. **Lisa X. Xu** and **Ping Liu**: Writing–review and editing.

## CONFLICT OF INTEREST STATEMENT

The authors declare no conflicts of interest.

## ETHICAL APPROVAL

All animal experiments were approved by the Animal Welfare Committee of Shanghai Jiao Tong University, and experimental methods were performed in accordance with the guidelines of Shanghai Jiao Tong University Animal Care (approved by Shanghai Jiao Tong University Scientific Ethics Committee, Registration No. 2020017).

## Supporting information



Supporting Information

## Data Availability

The datasets used and/or analysed during the current study are available from the corresponding author on reasonable request.
