# Supplementary material for: Reprogrammed MDSCs promote Th1‐dominant antitumour response via CD40 induced by autocrine TNF‐α after combining cryo‐thermal therapy with IL6 and IL17A neutralization
Source: Clin Transl Med. 2025 Oct 13;15(10):e70493. doi: 10.1002/ctm2.70493 (PMC12518503; doi:10.1002/ctm2.70493)
Supplement: Supplementary file 1 — Supporting Information [file CTM2-15-e70493-s001.docx]

**Supplementary Material**


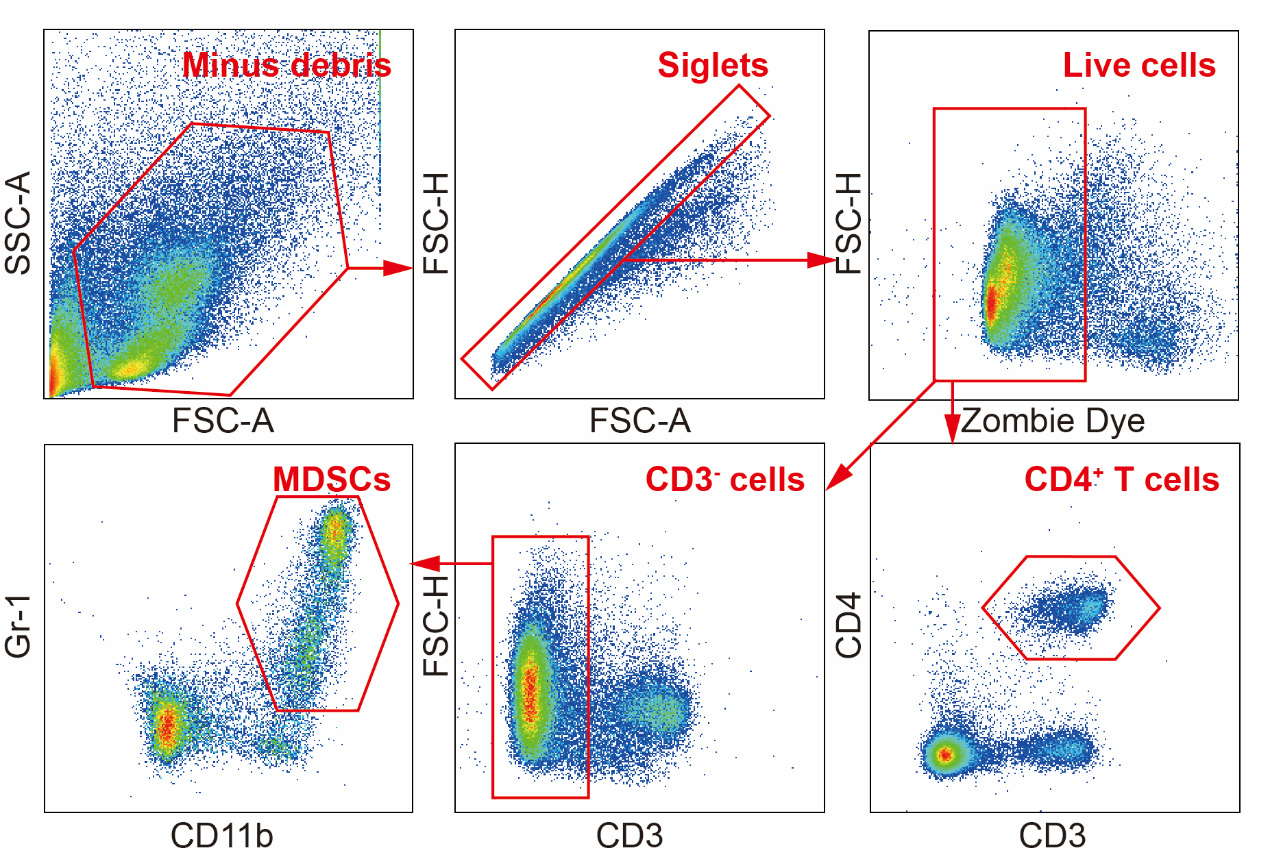


Figure S1. Gating strategy of CD4^+^ T cells and MDSCs of flow cytometry.


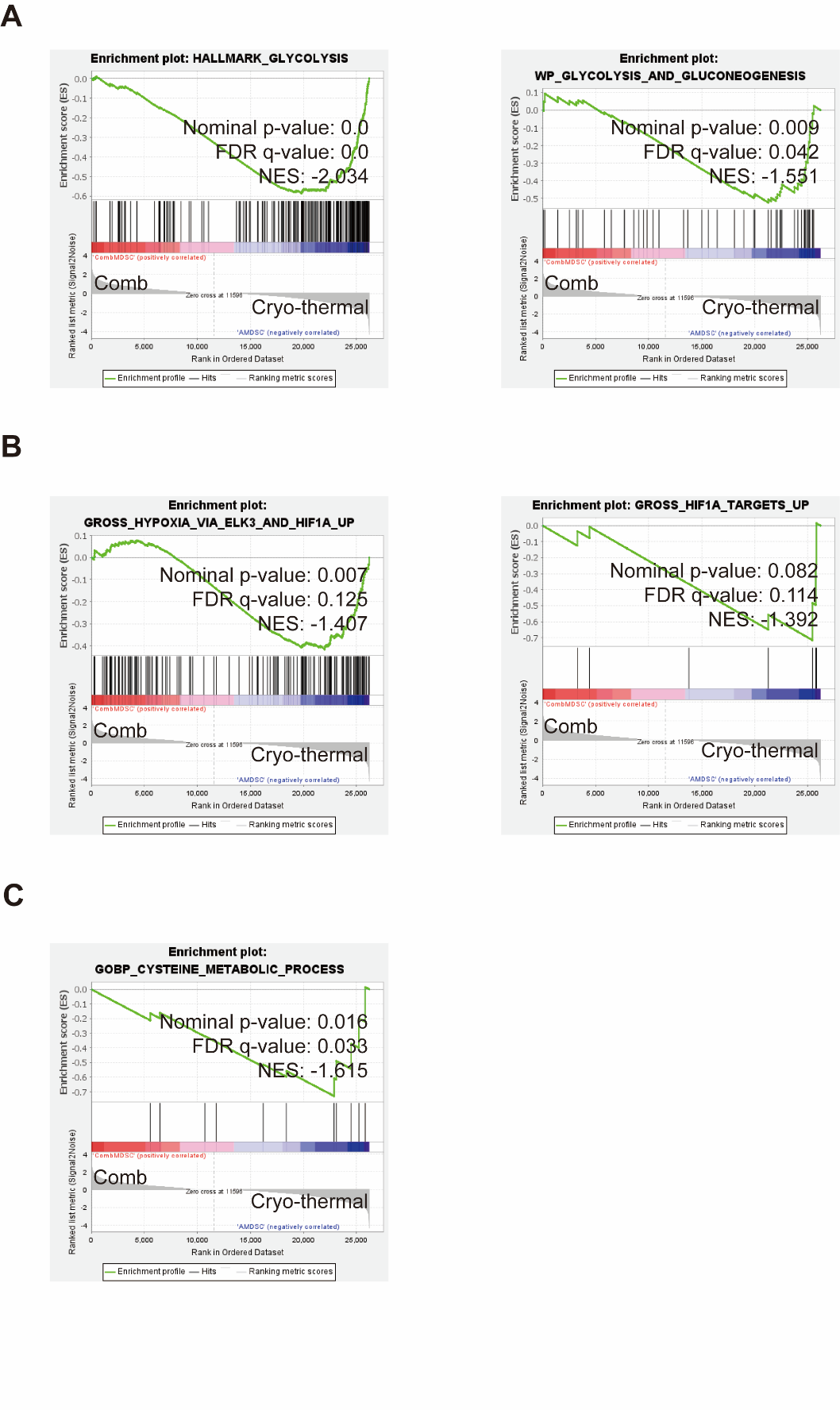


Figure S2. Gene set enrichment analysis of pathways of metabolic reprogramming in MDSCs. (A) Gene set enrichment analysis of Glucose metabolism pathways of MDSCs after combination therapy compared with cryo-thermal therapy. (B) Gene set enrichment analysis of Hypoxia-inducible factor 1α pathways of MDSCs after combination therapy compared with cryo-thermal therapy. (C) Gene set enrichment analysis of Cysteine metabolism of MDSCs after combination therapy compared with cryo-thermal therapy.


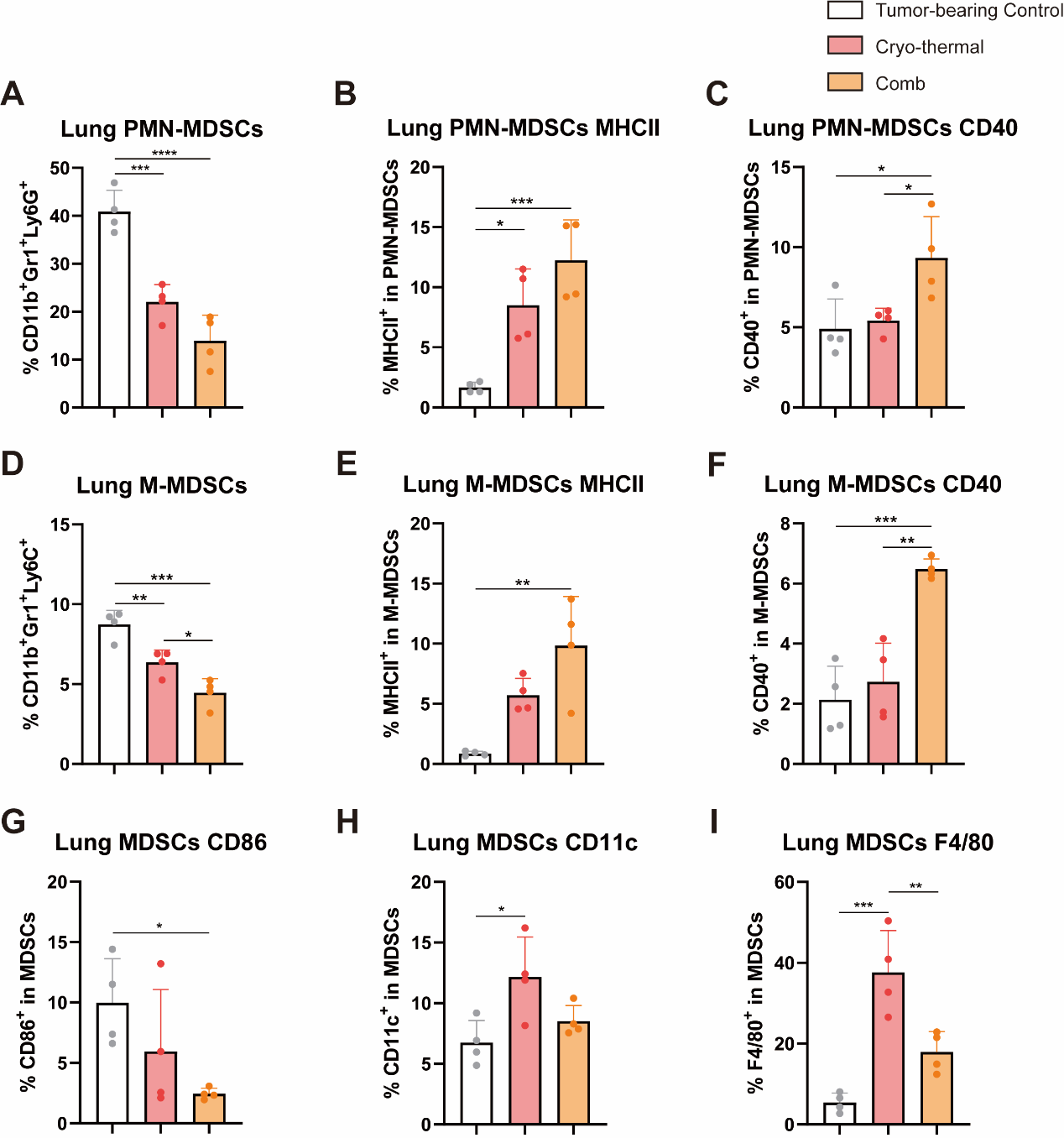


Figure S3. The proportion and mature markers of MDSCs. (A) The proportion of PMN-MDSCs. (B) The expression levels of MHCII on PMN-MDSCs. (C) The expression levels of CD40 on PMN-MDSCs. (D) The proportion of M-MDSCs. (E) The expression levels of MHCII on M-MDSCs. (F) The expression levels of CD40 on M-MDSCs. The expression levels of CD86 (G), CD11c (H), and F4/80 (I) on MDSCs. *p <0.05, **p<0.01, ***p <0.001. n = 4 for each group.


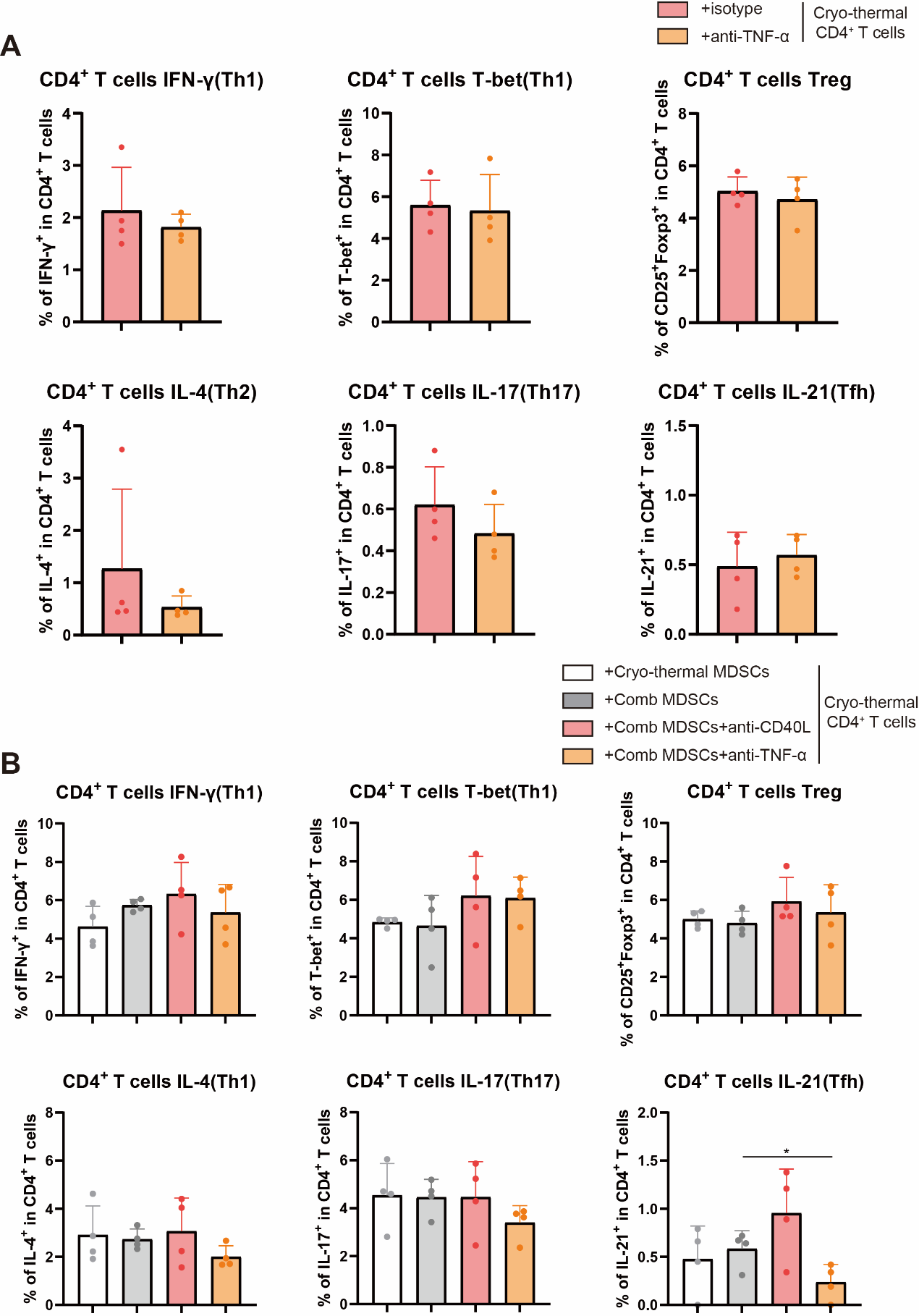


Figure S4. The direct effect of TNF-α on the differentiation of CD4^+^ T cells. (A) The subsets of CD4^+^ T cells individually cultured with anti-TNF-α. (B) The subsets of CD4^+^ T cells individually cultured with recombinant TNF-α. *p <0.05. n = 4 for each group.


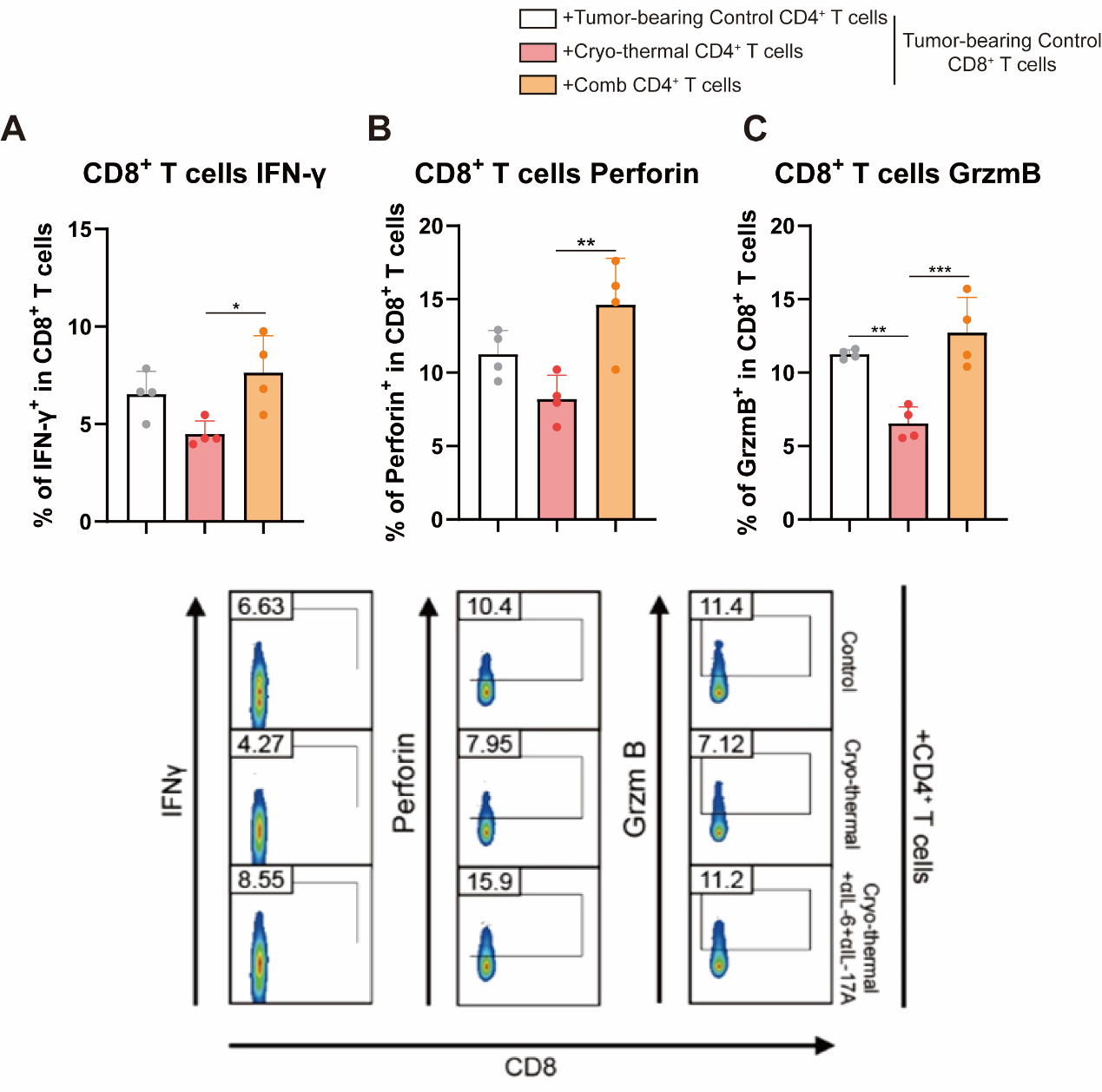


Figure S5. The effector function of CD8^+^ T cells co-cultured with CD4^+^ T cells from Tumor-bearing Control, Cryo-thermal and Combination therapy groups at 1:1 ratio. (A) The expression levels of IFN-γ in CD8^+^ T cells. (B) The expression levels of Perforin in CD8^+^ T cells. (C) The expression levels of Granzyme B in CD8^+^ T cells. *p <0.05, **p<0.01, ***p <0.001. n = 4 for each group.


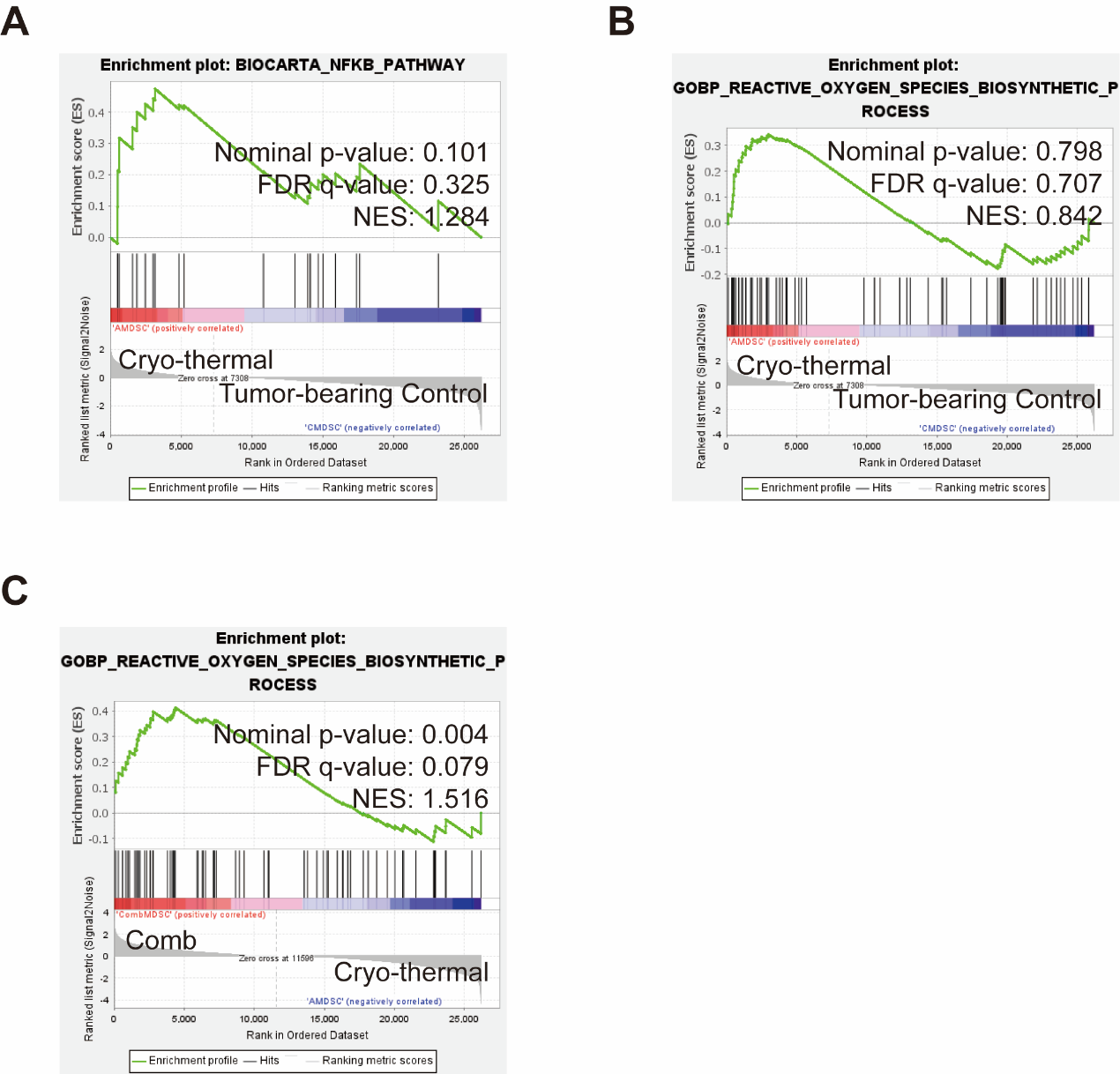


Figure S6. Gene set enrichment analysis of NF-κB pathway and Reactive oxygen species biosynthetic process. (A) Gene set enrichment analysis of NF-κB pathway of MDSCs after cryo-thermal therapy compared with Tumor-bearing Control. (B) Gene set enrichment analysis of Reactive oxygen species biosynthetic process of MDSCs after cryo-thermal therapy compared with Tumor-bearing Control. (C) Gene set enrichment analysis of Reactive oxygen species biosynthetic process of MDSCs after combination therapy compared with cryo-thermal therapy.


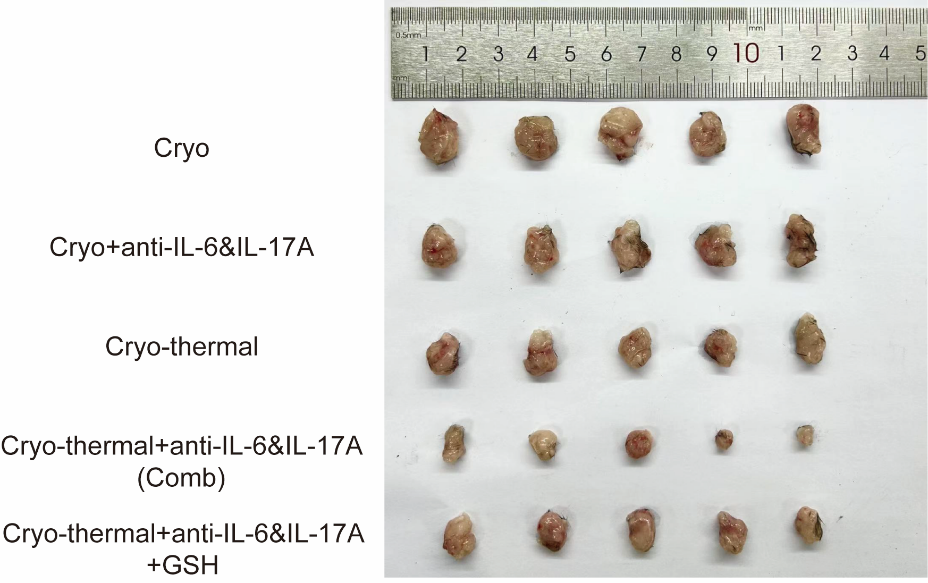


Figure S7. The photograph of untreated side tumor of mice was taken at the endpoint (n = 5/group).
